# Enhancing acidic oxygen evolution reaction with a highly stable Eu-doped RuO_2_ electrocatalyst

**DOI:** 10.1039/d6sc01665k

**Published:** 2026-08-07

**Authors:** Yin Qin, Kang Jie Li, Zilin Yan, Zhehan Ying, Kaikai Li, Hao Xiang Li, Tong-Yi Zhang

**Affiliations:** a Institute of Interdisciplinary Innovation in Low Carbon Metallurgical Engineering, School of Materials and Energy, Guangdong University of Technology Guangzhou 510006 China; b School of Science, School of Materials Science and Engineering, Harbin Institute of Technology Shenzhen 518055 China; c Materials Genome Institute, Shanghai University 333 Nanchen Road Shanghai 200444 China; d Guangzhou Municipal Key Laboratory of Materials Informatics, Advanced Materials Thrust, and Sustainable Energy and Environment Thrust, The Hong Kong University of Science and Technology (Guangzhou) Nansha Guangzhou 511400 Guangdong China; e Material Characterization and Preparation Facility, The Hong Kong University of Science and Technology (Guangzhou) Nansha Guangzhou 511400 Guangdong China

## Abstract

Ruthenium oxides (RuO_2_) are attractive alternatives for the oxygen evolution reaction (OER), but their stability in acidic environments is compromised by Ru oxidation and dissociation. Here, we leverage rare-earth (RE) europium (Eu) doping to engineer an Eu-RuO_2_ catalyst, exploiting the unique 4f orbital properties of Eu to modulate RE(f)–O(p)–Ru(d) orbital coupling for enhanced OER performance in acidic solution. The Eu-RuO_2_ catalyst exhibits a low overpotential of 195 mV, achieves 10 mA cm^−2^ current density, and exhibits outstanding stability for 2800 hours in 0.5 M H_2_SO_4_. In acidic PEM-WE devices, our Eu-RuO_2_ catalyst achieves a high current density of 1000 mA cm^−2^ at 1.67 V cell voltage and sustains operation at 1000 mA cm^−2^ at 60 °C in normal ambiance with negligible degradation for 300 hours. Density functional theory (DFT) calculations and *in situ* X-ray absorption spectroscopy (XAS) reveal that the 4f buffer band of Eu donates electrons to stabilize Ru–O covalency *via* 4f-2p-3d gradient orbital coupling, suppressing Ru oxidation and dissociation during acidic OER. Attenuated total reflection-surface-enhanced infrared absorption spectroscopy (ATR-SEIRAS) and DFT further show that this orbital coupling optimizes oxygen intermediate adsorption, lowering reaction barriers and stabilizing the acidic OER. Importantly, ATR-SEIRAS and DFT also confirm that Eu doping enhances the oxide path mechanism (OPM), evidenced by an O–O vibrational peak at low overpotential and a reduced rate-determining barrier. This kinetic advantage of the OPM, together with the effective suppression of Ru–O covalency loss, collectively explains the exceptional activity and long-term stability of the Eu-RuO_2_ catalyst under acidic conditions.

## Abstract 2

Eu's 4f buffer band stabilizes the Ru–O covalent bond through 4f–2p–3d gradient orbital coupling, thereby suppressing Ru oxidation and dissolution in PEM-WE.

## Graphical abstract

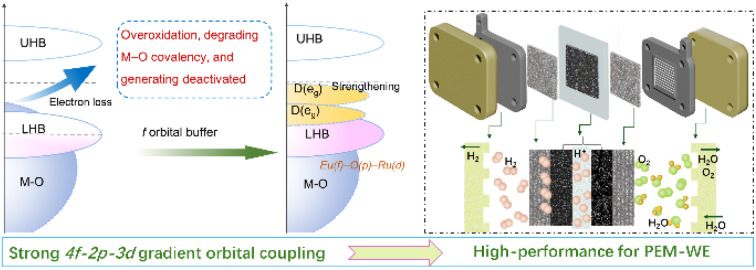

## Introduction

PEM-WE stands out as the most promising technology for large-scale green hydrogen production, owing to its high operational current density (>500 mA cm^−2^), ultrapure hydrogen output (>99.99%), and high-pressure compatibility (up to 80 bar).^1,2^ The sluggish OER in acidic solution remains the primary bottleneck for the commercialization of PEM-WE.^3,4^ These acidic limitations require electrocatalysts with exceptional long-term chemical stability to resist corrosion and degradation.^4,5^ Currently, IrO_2_ remains the dominant anode catalyst in PEM-WE, achieving an optimal balance between OER activity and operational durability.^6,7^ However, the extreme scarcity of Ir (crustal abundance: 0.001 ppm) poses an intrinsic scalability barrier for the PEM-WE technology, with current consumption rates predicted to deplete terrestrial reserves within 30 years.^8,9^ This critical supply-demand imbalance has driven systematic investigations into alternative catalysts. Among these, Ru-based oxides exhibit superior promise due to their unique electronic configurations, which enable efficient water dissociation.^10^ Nevertheless, the operational instability of Ru-based anodes arising from the progressive oxidation of Ru to soluble RuO_4_ at acidic OER potentials exceeding 1.4 V *vs.* RHE remains a critical limitation.^11–13^ While advanced modification strategies (*e.g.*, alloying and defect engineering) have improved catalyst stability, their long-term durability under industrial operating conditions (>500 mA cm^−2^) remains unsatisfactory.^14–17^

In recent years, RE elements such as La- and Er-doped RuO_2_ have significantly enhanced the acidic OER stability by suppressing lattice oxygen participation.^18,19^ We synthesized Eu-doped RuO_2_ (Eu-RuO_2_) to investigate 4f-mediated RE(f)–O(p)–TM(d) orbital coupling. Eu^3+^ possesses a unique half-filled 4f^7^ configuration, the lowest third ionization energy among REs, and an ionic radius well matched to Ru^4+^, inducing moderate lattice distortion without phase transformation. The Eu-RuO_2_ catalyst achieves exceptional performance, achieving a low overpotential of 195 mV and retaining stable activity for over 2800 h at 10 mA cm^−2^ at room temperature in normal ambiance. Moreover, when integrated into a PEM-WE, the Eu-RuO_2_-based system achieves 1000 mA cm^−2^ at a cell voltage of 1.67 V and demonstrates remarkable long-term stability at 1000 mA cm^−2^ for over 300 h at 60 °C in normal ambiance with negligible degradation. To deepen our understanding of the excellent performance, we follow the pioneering work on the RE(f)–O(p)–TM(d) gradient orbital coupling mechanism, published in 2023 by Fu *et al.*,^20^ the details of which are provided in the SI, where they investigated rare-earth (RE)-based transition metal (TM) oxides as OER electrocatalysts. The symmetry-adapted linear combination theory indicates that the 4f orbitals of RE elements can hybridize with O 2p orbitals *via* σ- and π-type bonding, which is particularly significant in the local *O*_h_ symmetry of the [TMO_6_] unit.^20,21^ Our characterization studies and DFT calculations reveal that electron donation from the Eu 4f buffer band preserves Ru–O covalency *via* gradient 4f–2p–3d orbital coupling. *In situ* XAS and DFT show that the Eu–O–Ru unit suppresses Ru–O bond breakage and Ru oxidation state increase during the OER. Notably, ATR-SEIRAS and DFT confirm that Eu doping enhances the OPM, evidenced by an O–O peak at low potential and a reduced rate-determining barrier. This OPM advantage, combined with the suppression of Ru–O covalency loss, explains the exceptional activity and stability of Eu-RuO_2_.

## Results

**Fig. 1:**
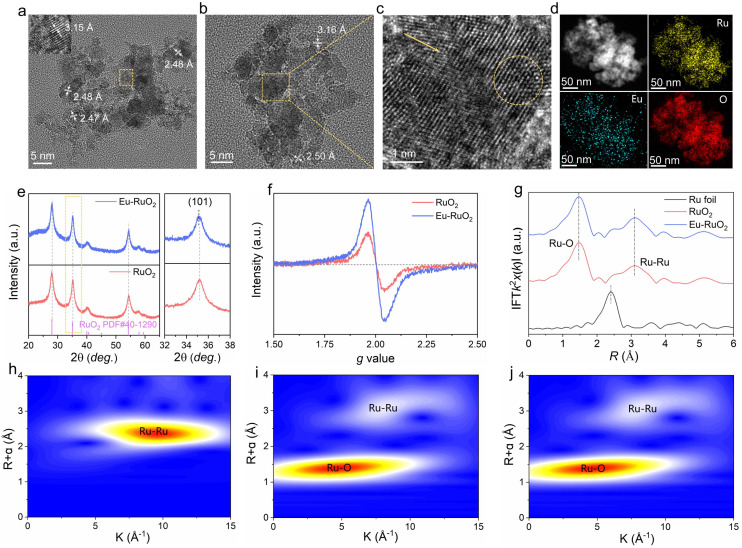
Structural and compositional characterization. (a) HR-TEM image of the RuO_2_ catalyst. (b and c) HR-TEM images of the Eu-RuO_2_ catalyst. (d) Elemental mapping of Eu, Ru, and O in the Eu-RuO_2_ catalyst. (e) XRD patterns with a local magnification of the (101) peak of RuO_2_ and Eu-RuO_2_. (f) ESR spectra of RuO_2_ and Eu-RuO_2_. (g) Fourier-transformed Ru K-edge EXAFS spectra of Ru foil, RuO_2_, and Eu-RuO_2_. (h–j) Corresponding WT for (h) Ru foil, (i) RuO_2_, and (j) Eu-RuO_2_.

The Eu-RuO_2_ catalyst was synthesized *via* a facile hydrothermal method (Fig. S1). Under high temperature and high pressure, ammonia water was strategically employed to establish a weakly alkaline environment, enabling aqueous co-precipitation of Ru^3+^ and Eu^3+^ ions, followed by annealing to achieve simultaneous morphological control and Eu^3+^ doping of RuO_2_ (see Methods). For comparison, pure RuO_2_ was prepared through an analogous procedure without the addition of Eu ions. Scanning electron microscopy (SEM) and transmission electron microscopy (TEM) images (Fig. S2 and S3) confirm that RuO_2_ and Eu-RuO_2_ exhibit similar morphologies. High-resolution TEM (HR-TEM) reveals that both RuO_2_ and Eu-RuO_2_ are comprised of sub-5 nm nanocrystallites, with Eu-RuO_2_ displaying lattice distortions and defects (Fig. 1a–c). Effective Eu-doping in Eu-RuO_2_ is directly resolved by time of flight secondary ion mass spectrometry (TOF-SIMS), which detects Eu fragment signals (Fig. S4) and verifies the homogeneous distribution of Ru, Eu, and O by elemental mapping analysis (Fig. 1d). To further resolve the atomic-scale distribution, we analyzed the atomic brightness contrast in HAADF-STEM images, where Eu atoms appear brighter than Ru atoms owing to their higher atomic mass (Fig. S5). Furthermore, the Eu-RuO_2_ powder sample was immersed in 0.5 M HCl for approximately 48 h. Subsequent SEM-EDS analysis of the residual solid (Fig. S6) persistently revealed a discernible Eu signal, whereas ICP-OES quantification of the corresponding leachate yielded no detectable Eu content, conclusively ruling out the presence of any acid-soluble Eu-containing secondary phases. HR-TEM analysis (Fig. 1a and b) further reveals the incorporation of Eu^3+^ into the RuO_2_ lattice, as evidenced by the expanded interplanar spacings of Eu-RuO_2_ (3.16 Å for (110) and 2.50 Å for (101)) compared to RuO_2_ (3.15 Å and 2.48 Å, respectively). This lattice expansion originates from the ionic radius mismatch between Eu^3+^ (0.947 Å) and Ru^4+^ (0.76 Å), where substitution of larger Eu^3+^ ions induces tensile strain within the rutile matrix. The downshift of the (101) diffraction peak for Eu-RuO_2_ in the XRD pattern (Fig. 1e) further demonstrates the lattice expansion due to Eu^3+^ doping. Notably, Eu-RuO_2_ exhibits a significantly enhanced electron spin resonance (ESR) signal at *g* = 2.003 compared to RuO_2_ (Fig. 1f), indicative of unpaired electrons associated with increased oxygen vacancies (V_o_) in Eu-RuO_2_, which is consistent with the HR-TEM observations.^20^

To elucidate the valence states and the unique electronic configuration of Eu-RuO_2_ electrocatalysts, X-ray photoelectron spectroscopy (XPS) analysis was performed on both Eu-RuO_2_ and RuO_2_ for comparative assessment. High-resolution XPS of the Ru 3d region (Fig. S7) resolves two spin–orbit doublets at 280.5 eV (Ru 3d_5/2_) and 284.7 eV (Ru 3d_3/2_), accompanied by satellite peaks for RuO_2_. Notably, the Ru 3d peaks of Eu-RuO_2_ exhibit a positive shift relative to those of RuO_2_ at both 280.55 eV (Ru 3d_5/2_) and 284.77 eV (Ru 3d_3/2_), implying electron withdrawal from Ru^4+^ and a higher oxidation state of Ru in Eu-RuO_2_.^22–24^ In the high-resolution O 1s XPS spectra (Fig. S8), the peaks at 528.8, 530.0, 531.0, and 532.1 eV correspond to lattice oxygen (O_lat_), oxygen vacancies (V_O_), surface hydroxyl groups (OH^−^), and adsorbed water molecules, respectively.^25,26^ Compared to pristine RuO_2_, the Ru–O bond peak in Eu-RuO_2_ exhibits a slight positive binding energy shift, consistent with the trend observed in the Ru 3d XPS results. Furthermore, the area ratio of lattice oxygen to oxygen vacancy peaks (O_lat_/V_O_) increases from 0.88 in RuO_2_ to 1.42 in Eu-RuO_2_. This significant change clearly demonstrates that Eu doping introduces abundant oxygen vacancies on the RuO_2_ surface. X-ray absorption fine structure (XAFS) spectroscopy further probed the oxidation state and local atomic structure of RuO_2_ and Eu-RuO_2_. The Ru K-edge extended X-ray absorption fine structure (EXAFS) spectra exhibit two characteristic peaks at 1.47 Å (Ru–O) and 3.19 Å (Ru–Ru) for RuO_2_(Fig. S9),^22,27,28^ with wavelet transform (WT) analysis confirming these bond assignments (Fig. 1g–j). Fitting of the Ru K-edge EXAFS spectra yielded coordination numbers (CNs) for Ru–O and Ru–Ru bonds in Eu-RuO_2_ of 6.3 and 10.0, respectively (Table S1). Compared to pristine RuO_2_ (Ru–O CN: 6.8; Ru–Ru CN: 11.2), the significant reduction in Ru–Ru coordination (ΔCN = −1.2), together with a moderate decrease in Ru–O coordination (ΔCN = −0.5), collectively confirms the formation of oxygen vacancies in Eu-RuO_2_. The observed Ru–O CN, which exceeds 6, likely arises from multiple-scattering effects and lattice distortions. The companion peak observed at approximately 2.7 Å serves as a characteristic indicator of the rutile-type structure in both RuO_2_ and Eu-RuO_2_. The spectra demonstrate a reduced Ru–O coordination number in Eu-RuO_2_ compared to RuO_2_, with the Ru–O bond length shortened and the Ru–Ru atomic distance increased upon Eu doping, all originating from Eu-induced symmetry disruption in the RuO_2_ lattice and indicating oxygen vacancy formation in Eu-RuO_2_.

**Fig. 2:**
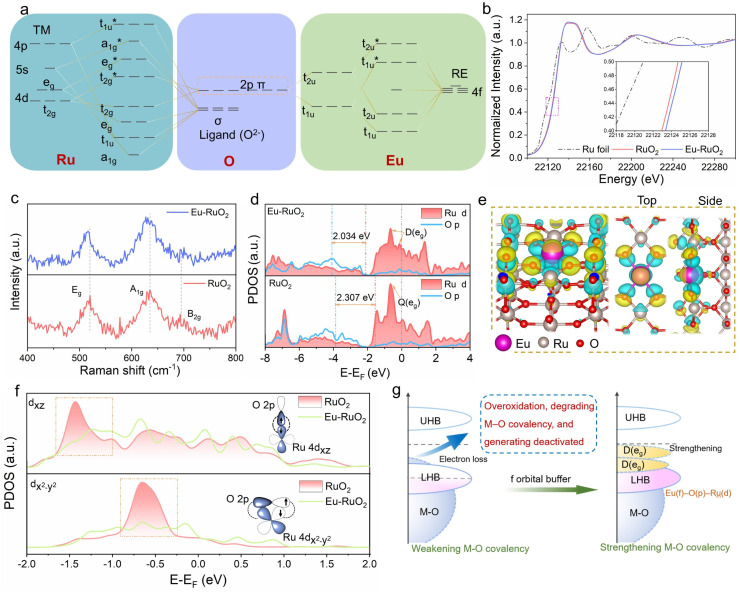
The strong coupling effect of Eu(4f)–O(2p)–Ru(3d). (a) Qualitative molecular orbital diagram derived from (TMO_6_) and (REO_6_) with *O*_h_ symmetry (theoretical model diagrams of RuO_2_ before (left) and after (right) Eu doping). (b) Ru K-edge XANES spectra of Ru foil, RuO_2_, and Eu-RuO_2_. (c) Raman spectra of RuO_2_ and Eu-RuO_2_. (d) PDOS of Ru d and O p of RuO_2_ and Eu-RuO_2_. (e) Charge density difference of Eu-RuO_2_ with different views. (f) The orbital-projected states of Ru d_*xz*_ and d_*x*^2^−*y*^2^_ in RuO_2_ and Eu-RuO_2_. (g) The electronic modulation effect of Eu6c within the Eu6c–O–Ru6c unit site is elucidated through the Mott–Hubbard model.

Guided by group theory-based symmetry analysis, TMOs exhibit structurally distinct TMO_6_ octahedral edge motifs adopting *O*_h_ point group symmetry (Fig. 2a and S10), which illustrate the orbital coupling patterns among the RE(f), O(p), and TM(d) orbitals.^20,21^ The introduction of RE elements into TMOs enables the construction of RE(f)–O(p)–TM(d) architectures, where ionically dominated RE–O bonds preserve the covalent TM–O network while facilitating charge compensation through electron extraction from the RE 4f states at higher energy.^20^ To elucidate the chemical coordination and valence electron redistribution at the Eu–O–Ru centers in Eu-RuO_2_, XAFS spectroscopy was used. Fig. 2b shows the Ru K-edge XANES spectra of Ru foil, RuO_2_, and Eu-RuO_2_. Prior to XAS measurements of RuO_2_ and Eu-RuO_2_ samples, energy calibration was performed using a Ru foil reference (Fig. S11). Comparative spectral examination reveals a positive shift in the absorption edge upon Eu doping (Fig. 2b), indicative of a slight increase in Ru's oxidation state and enhanced coupling between Ru and O. This valence state modification is further corroborated by the enhanced white-line intensity at the Ru K-edge (Fig. S12),^4^ consistent with XPS (Fig. S7 and 8) and Bader charge calculations of Ru and O (Fig. S13 and 14). These collective observations provide evidence partial electron occupancy in Ru d-orbitals arising from charge redistribution within the Eu–O–Ru coordination framework. Raman spectroscopy deconvolution (Fig. 2c) confirms the nanocrystalline rutile-phase RuO_2_ structure *via* characteristic phonon modes at 519.98 cm^−1^ (E_g_, Ru–O bending), 634.58 cm^−1^ (A_1g_, Ru–O stretching), and 700 cm^−1^ (B_2g_, O–Ru–O scissoring). The Raman spectral broadening and blue shift in the A_1g_ mode are attributed to phonon softening from reduced local symmetry and increased oxygen vacancies due to Eu doping.^29,30^ The projected density of states (PDOS) elucidates the electronic modulation induced by Eu(f)–O(p)–Ru(d) orbital coupling in Eu-doped RuO_2_ (Fig. 2d), revealing a systematic downshifting of both Ru d-band and O p-band centers accompanied by a reduced energy separation (Δ*E* = 2.034 eV *vs.* 2.307 eV in RuO_2_),^31^ which directly correlates with strengthened Ru–O covalency through rehybridized bonding orbitals. This finding is further supported by charge density distribution diagrams of RuO_2_ and Eu-RuO_2_ (Fig. S15). Charge density differentials (Fig. 2e) reveal pronounced electron density depletion at Eu^3+^ centers, contrasting with the partial charge retention observed at adjacent oxygen ligands. These results stem from the strong electronegativity gradient within the Eu–O–Ru triad, where oxygen maintains electron-accepting character.

DFT calculations for the Eu-RuO_2_ systems (Eu outermost electron configuration: 4f^7^ 6s^2^) reveal distinct thermodynamic preferences (Fig. S16): Eu^2+^ incorporated configurations exhibit elevated total energies (−1027.24 eV relative to the RuO_2_ baseline of −1030.60 eV), whereas Eu^3+^ doping induces significant system stabilization with a 3.36 eV energy reduction (−1033.72 eV), illustrating the crucial role of 4f electrons in stabilizing RuO_2_. The modulation effects of Eu 4f induce additional splitting of the Q(e_g_) state into two double-degenerate D(e_g_) states, enhancing electronic distribution around E_F_ through increased contributions from d_*xz*_ and d_*x*^2^−*y*^2^_ states (Fig. 2d, f and S17). Within the optimized Eu6c–O–Ru6c unit site, the Eu 4f state is positioned above E_F_, underscoring its electron-donating capacity.^20^ In RuO_2_, the LHB's location above the M−O band results in a smaller Hubbard U relative to Δ,^32^ weakening interactions between Ru6c sites and oxygen adsorbates due to reduced electronic density. Incorporating Eu6c into the Eu6c–O–Ru6c unit site offsets LHB-related electron loss, stabilizing M–O bonds and promoting D(e_g_) state electrons closer to E_F_*via* lattice strain-induced symmetry disruption (Fig. 2g). For O_2_'s triplet ground state (π^2^p*↑↑), d_*xz*_ and d_*x*^2^−*y*^2^_ orbitals facilitate O_2_ formation during the OER by interacting with oxygen adsorbates through σ and π bonds, offering a spin-selective stabilization effect.^20^

**Fig. 3:**
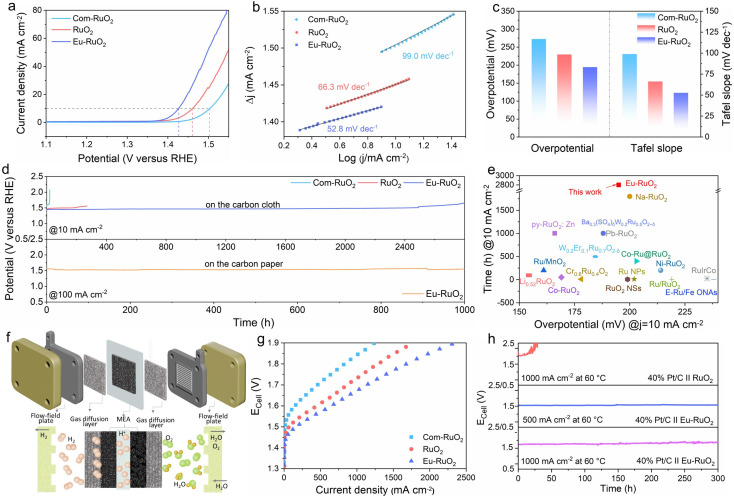
OER and PEM-WE performance. (a) The polarization curves tested at a scan rate of 5 mV s^−1^. (b) Tafel plots. (c) The comparison of overpotentials at 10 mA cm^−2^ and Tafel slopes of commercial RuO_2_ (Com-RuO_2_), synthesized RuO_2_, and Eu-RuO_2_. (d) Chronopotentiometry curve of Com-RuO_2_, synthesized RuO_2_ and Eu-RuO_2_ at a current density of 10 mA cm^−2^. (e) Performance comparison of noble-metal-based OER catalysts in acidic electrolyte.^14,22,33–46^ (f) Schematic diagram of the PEM-WE device structure. (g) Polarization curve of the PEM-WE electrolyzer. (h) Chronopotentiometry curves of Com-RuO_2_, synthesized RuO_2_ and Eu-RuO_2_ in the PEM electrolyzer.

The OER performance of Eu-RuO_2_ and synthesized RuO_2_ was evaluated in an oxygen-saturated 0.5 M H_2_SO_4_ solution using a three-electrode system with a carbon rod counter electrode and Ag/AgCl reference electrode at room temperature in normal ambiance.^33^ For comparison, a commercial RuO_2_ electrocatalyst was also examined under identical conditions. As shown in Fig. 3a, the linear sweep voltammetry (LSV) curves indicate that Eu-RuO_2_ exhibits superior OER activity (the fluctuations in the polarization curves are attributed to bubbles or external environmental factors), requiring only 195 mV overpotential to reach a current density of 10 mA cm^−2^ (*η*_10_), outperforming synthesized RuO_2_ (230 mV) and commercial RuO_2_ (273 mV). We have further extended our investigation to include the synthesis and electrochemical evaluation of Ce- and Sm-doped RuO_2_ catalysts (Fig. S18 and 19). As presented in Fig. S20 and 21, all rare-earth-metal (Eu, Sm, Ce) doped RuO_2_ catalysts show superior OER activity compared to pristine RuO_2_ commercial IrO_2_, and commercial RuO_2_, following the order Eu-RuO_2_> Ce-RuO_2_ > Sm-RuO_2_. These results further highlight the crucial role of the f–p–d coupling mechanism in boosting OER performance, with Eu doping playing a particularly important role. The Tafel slope of Eu-RuO_2_ (52.8 mV dec^−1^) is substantially smaller than those of synthesized RuO_2_ (66.3 mV dec^−1^) and commercial RuO_2_ (99.0 mV dec^−1^) (Fig. 3b and c), indicating that optimized Eu doping enhances OER kinetics and electron transfer efficiency.^34^ To evaluate the intrinsic activity per active site of Eu-RuO_2_, synthesized RuO_2_, and commercial RuO_2_, electrochemical specific surface area (ECSA) was estimated by determining the electrochemical double-layer capacitance (*C*_dl_), which was derived from cyclic voltammetry in the non-faradaic region (Fig. S22–24). ECSA-normalized LSV analysis in Fig. S25 demonstrates that the intrinsic activity improvement arises from Eu incorporation rather than surface area effects. Furthermore, the LSV curves of Com-RuO_2_, RuO_2_, and Eu-RuO_2_ were further normalized by Ru mass loading, and the results are presented in Fig. S26. Notably, the intrinsic activity of RuO_2_ is substantially enhanced upon Eu doping, confirming that RE(f)–O(p)–Ru(d) orbital coupling effectively modulates the electronic structure of RuO_2_. This conclusion is further corroborated by a comparison of the apparent TOF values measured at overpotentials of 230 mV and 250 mV (Fig. S27).

In addition to electrochemical activity, long-term stability is paramount for OER electrocatalysts in industrial applications. The stability of the Eu-RuO_2_ and control RuO_2_ catalysts was first evaluated by a chronopotentiometry test at 10 mA cm^−2^ on carbon cloth. While our synthesized RuO_2_ exhibited better stability than commercial RuO_2_ (which degraded within 15 h), its performance still declined rapidly, sustaining operation for only 250 h (Fig. 3d). Strikingly, the Eu-RuO_2_ catalyst retained its initial current density for over 2800 hours under identical testing conditions, representing an unprecedented benchmark for acidic OER electrocatalysis and surpassing all recently reported noble metal-based catalysts (Fig. 3e and Table S2).^14,22,33–46^ Furthermore, we replaced the substrate with carbon paper and conducted additional stability tests at a high current density of 100 mA cm^−2^, and the results show that Eu-RuO_2_ can operate stably for more than 1000 hours, further demonstrating its outstanding stability (Fig. 3d). We characterized the Eu-RuO_2_ catalyst after OER testing using HR-TEM imaging and corresponding EDS mapping. The HRTEM images reveal that the morphology of Eu-RuO_2_ remains largely unchanged after the reaction; although the surface of the crystal structure appears slightly blurred, the lattice fringes remain clearly visible (Fig. S28). The results show that the Ru, Eu, and O elements remain uniformly distributed, and a distinct Eu signal is still detectable (Fig. S29). And ICP analysis of the electrolyte collected after OER testing showed a Ru dissolution amount of 52 ppb, with the Eu signal remaining below the detection limit. Collectively, these results demonstrate the excellent crystalline structural stability of Eu-RuO_2_.

Eu-RuO_2_, with superior activity (*η*_10_ = 195 mV) and durability (>2800 h at 10 mA cm^−2^) in three-electrode configurations, was further assessed for its industrial performance in a PEM-WE, where the tested OER catalyst served as the OER anode at 60 °C in normal ambiance, paired with a commercial Pt/C HER cathode (40 wt% Pt) and separated by a Nafion 115 membrane (Fig. 3f). At a 1000 mA cm^−2^ testing current density, the operational voltages are 1.84 V for commercial RuO_2_, 1.73 V for synthesized RuO_2_ and only 1.67 V for Eu-RuO_2_ (Fig. 3g), highlighting the superior electrochemical efficiency of Eu-RuO_2_ over both commercial and synthesized RuO_2_. Remarkably, the Eu-RuO_2_‖Pt/C PEM-WE demonstrated zero degradation during 300-hour continuous operation at 1000 mA cm^−2^ (Fig. 3h), starkly contrasting with the synthesized RuO_2_‖Pt/C PEM-WE, which failed after only 15 hours under identical conditions. Furthermore, the Eu-RuO_2_‖Pt/C PEM-WE maintained stable operation at 500 mA cm^−2^ for 300 hours, highlighting its exceptional durability for practical green hydrogen production.

**Fig. 4:**
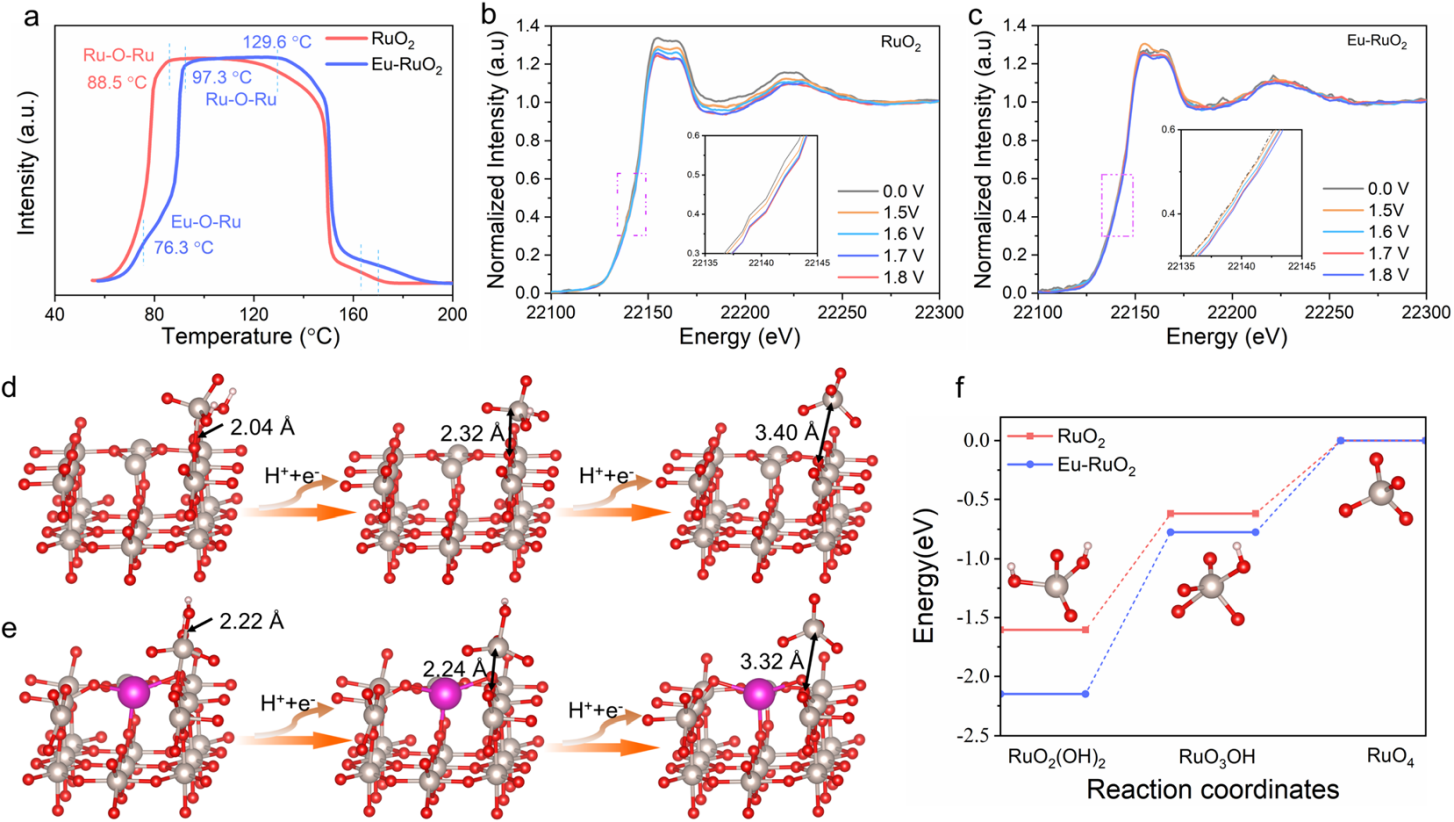
Enhancement of OER stability. (a) H_2_-TPR spectra of RuO_2_ and Eu-RuO_2_. (b and c) Ru K-edge XANES spectra of RuO_2_ (b) and Eu-RuO_2_ (c) with different applied biases. (d and e) Illustration of Ru dissolution at the step edge of RuO_2_ and Eu-RuO_2_(110) surfaces, showing the sequential oxidation of the Ru dissolution intermediate RuO_2_(OH)_2_ for RuO_2_ (d) and Eu-RuO_2_ (e). (f) Potential energy landscape during two sequential deprotonations, revealing that Ru dissolution from Eu-RuO_2_ requires higher energy than that from RuO_2_.

Hydrogen temperature-programmed reduction (H_2_-TPR) analyses (Fig. 4a) reveal distinct thermodynamic stabilization mechanisms between the catalysts. RuO_2_ demonstrates a characteristic reduction event at 88.5 °C, corresponding to the irreversible phase transition from Ru^4+^ to metallic Ru^0^,^29^ while Eu-RuO_2_ exhibits a 9.9% upward shift in primary reduction temperature (97.3 °C), indicative of enhanced oxidative state stability through Eu doping. The morphological similarity (Fig. 1a and b) excludes size-dependent effects, confirming the intrinsic stabilization mechanism. Notably, the emergence of a secondary reduction feature at 76.3 °C in Eu-RuO_2_ correlates with the Ru–O–Eu asymmetric coordination environments.^29^*Operando* Ru K-edge XANES spectroscopy was performed at applied potentials ranging from 1.5 to 1.8 V *vs.* RHE to investigate potential-dependent structural evolution, with particular emphasis on the dynamics of the Ru oxidation state and alterations in the local coordination environment (Fig. S30). As evidenced by the differential edge energy shift profiles in Fig. 4b and c, Eu-RuO_2_ and RuO_2_ exhibit distinct evolution patterns under electrochemical polarization. The near-edge absorption curve of RuO_2_ shows a more pronounced increase with rising potential compared to that of Eu-RuO_2_, thus evidencing the superior crystalline stability of the Eu-stabilized architecture. Furthermore, *in situ* Raman spectroscopy of Eu-RuO_2_ reveals that the E_9_ peak position remains unchanged with increasing voltage, providing additional evidence for the excellent structural stability of Eu-RuO_2_ (Fig. S31). We further conducted a systematic evaluation of the energy changes associated with surface Ru dissolution and lattice oxygen loss from the surfaces of both RuO_2_ and Eu-RuO_2_ (110). The proposed process of surface Ru dissolution and lattice oxygen loss is depicted in Fig. S32 and 33, where Ru at the labeled Ru and oxygen sites is displaced from the (110) surface. Our results reveal that lattice-incorporated Eu significantly enhances the Ru dissolution energy barrier from 1.40 eV (pure RuO_2_) to 1.96 eV (Eu-RuO_2_). Concurrently, subsurface oxygen removal requires 2.69 eV for RuO_2_*versus* 2.85 eV for Eu-RuO_2_, quantitatively confirming the reinforced structural stability of the Eu-modified system. This stabilization originates from the synergistic Eu(f)–O(p)–Ru(d) orbital hybridization, which strengthens interfacial bonding and suppresses surface degradation pathways.

Numerous studies have reported that the OER process of RuO_2_ involves the formation of high-valent Ru clusters,^47,48^ such as the mechanism proposed by Klyukin *et al.*, where Ru dissolution occurs *via* deprotonation of the RuO_2_(OH)_2_ cluster. Building on this understanding, we conducted calculations to evaluate the energetics of the dissolution process (Fig. 4d–f). In these calculations, Ru atoms were removed from unsaturated surface coordination sites (with identical positions maintained across all models), and O atoms were sourced from the surrounding environment of these Ru sites. On Eu-RuO_2_(110), the removal of two surface hydrogen atoms for the deprotonation of RuO_2_(OH)_2_ (m1) to RuO_3_OH (m2) requires an activation free energy of 1.38 eV, which is 0.40 eV higher than that on RuO_2_(110) (0.98 eV). Specifically, the RuO_3_OH intermediate forms a chemical bond with m1–O, with a Ru–O bond length of 2.22 Å in Eu-RuO_2_, which elongates to 2.24 Å for m2–O. In contrast, in RuO_2_, the corresponding bond lengths are 2.04 Å for m1–O and 2.32 Å for m2–O (Fig. 4d–f). Additionally, the second deprotonation leading to RuO_4_ dissolution requires an activation free energy of 2.15 eV on Eu-RuO_2_(110), which is 0.55 eV higher than that on RuO_2_(110) (1.6 eV). Notably, the m3–O bond length in Eu-RuO_2_ was found to be 3.32 Å, shorter than that in RuO_2_ (3.40 Å). These structural and energetic differences suggest that the strong binding of the Ru dissolution intermediate on Eu-RuO_2_ enhances the catalyst's durability under operational conditions, but no analogous stabilization occurs in RuO_2_.^33^

**Fig. 5:**
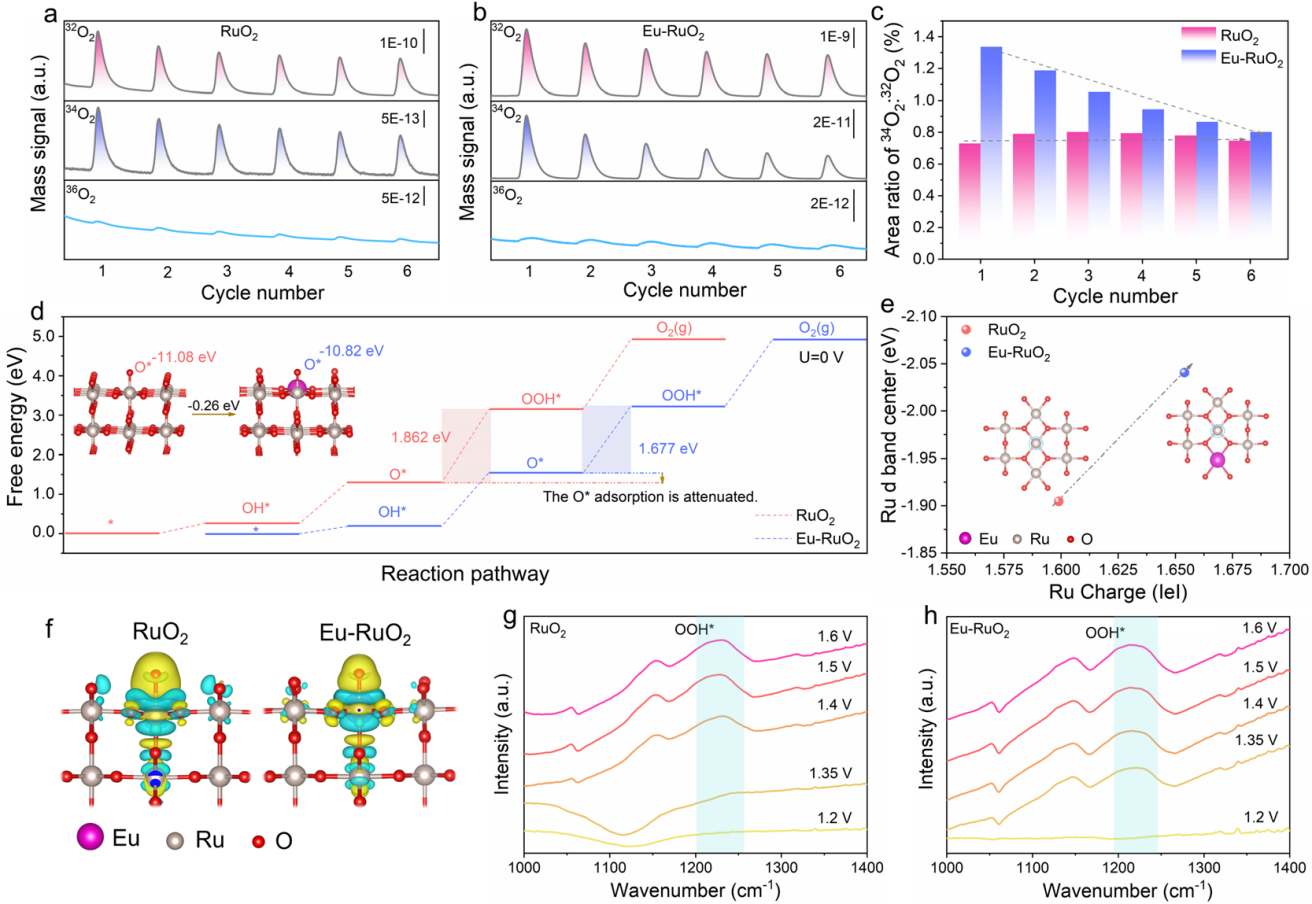
OER mechanism. (a and b) The DEMS signals of ^32^O_2_, ^34^O_2_, and ^36^O_2_ evolution measured during three consecutive cycles in ^16^O-enriched H_2_SO_4_ electrolyte (H_2_^16^O), revealing distinct oxygen exchange behaviors between the ^18^O-labeled catalysts (denoted as RuO_2_ (a) and Eu-RuO_2_ (b)). (c) The ^34^O_2_/^32^O_2_ peak area ratio derived from mass spectroscopy (MS) distinctly differentiates the oxygen exchange dynamics between RuO_2_ and Eu-RuO_2_. (d) Gibbs free energy diagrams of RuO_2_ and Eu-RuO_2_. (e) The Ru d-band centers of RuO_2_ and Eu-RuO_2_ are shown with shifts in Ru charge. The inset provides the Ru charge values for RuO_2_ and Eu-RuO_2_, determined *via* Bader charge analysis. (f) Charge density difference of *O-adsorbed RuO_2_ (left) and Eu-RuO_2_ (right). (g and h) ATR-SEIRAS analysis of RuO_2_ (g) and Eu-RuO_2_ (h).

Isotopic ^18^O labeling coupled with *in situ* differential electrochemical mass spectroscopy (DEMS) was utilized to investigate the lattice oxygen participation in acidic OER for various catalysts (Fig. 5a–c). Catalysts were labeled with ^18^O *via* cyclic voltammetry (CV) in a 0.5 M H_2_SO_4_/H_2_^18^O solution, sweeping potentials from 1.00 to 1.55 V *versus* RHE at a rate of 5 mV s^−1^. Subsequent DEMS analysis during the OER in a 0.5 M H_2_SO_4_/H_2_^16^O solution revealed that the majority of evolved O_2_ from both RuO_2_ and Eu-RuO_2_ corresponded to ^32^O_2_ (Fig. 5a–c), indicating that the dominant reaction pathway for both catalysts does not involve the lattice oxygen mechanism (LOM). The ^34^O_2_/^32^O_2_ ratios for both catalysts exceeded the natural abundance of H_2_^18^O (0.2%), indicating partial lattice oxygen participation in both catalysts.^19,49^ Notably, Eu-RuO_2_ initially exhibits a higher ^34^O_2_/^32^O_2_ ratio than RuO_2_, but this ratio decreases significantly with testing time, whereas RuO_2_'s ratio of ^34^O_2_/^32^O_2_ remains stable. This observation may be attributed to the higher concentration of oxygen vacancies in Eu-RuO_2_, which could enhance its affinity for ^18^O.

To elucidate the mechanism underlying the enhanced electrocatalytic activity of Eu-RuO_2_ in the OER, we conducted systematic DFT simulations by constructing atomic-scale models of both RuO_2_ and Eu-RuO_2_ (Fig. S34–36). Thermodynamic profiles of oxygen-containing intermediates along the AEM pathway were evaluated to unravel the origin of catalytic enhancement, with particular emphasis on Gibbs free energy calculations that corroborate DEMS observations. Computational results reveal that the rate-determining step (RDS) for both catalysts is the transformation of adsorbed oxygen species (*O) to hydroperoxyl intermediates (*OOH) (Fig. 5d). Notably, Eu-RuO_2_ exhibits a substantially lower activation energy barrier (Δ*G* = 1.677 eV) compared to RuO_2_ (Δ*G* = 1.862 eV), suggesting that Eu incorporation effectively modulates the activity of active sites. This enhancement is driven by optimized *O adsorption, where DFT calculations reveal a more negative adsorption energy for Eu-RuO_2_ (−10.82 eV) *versus* RuO_2_ (−11.08 eV). The downshifted Ru d-band center in Eu-RuO_2_ weakens interactions between Ru active sites and oxygen intermediates, thereby reducing the energy barrier of the RDS. Bader charge analysis further elucidates that Eu (4f)–O (2p)–Ru (3d) orbital coupling within Eu-RuO_2_ facilitates interfacial charge redistribution, increasing the Ru oxidation state (Fig. 5d and S13, 14), ultimately achieving a low overpotential of 195 mV in the OER. Spatial charge density difference analysis (Fig. 5f and S37) demonstrates diminished electron density accumulation proximal to the adsorbed intermediate, revealing attenuated electronic coupling within the Eu–O–Ru catalytic triad and the chemisorbed intermediates due to Eu incorporation. To elucidate the dynamic evolution of oxygen-containing intermediates during the OER, *operando* ATR-SEIRAS investigations were conducted using a customized three-electrode spectroelectrochemical cell with a Si prismatic internal reflection element (Fig. S38). Spectral acquisition spanning 1000–1400 cm^−1^ was synchronized with LSV across the potential window of 1.2–1.6 V *vs.* RHE. As demonstrated in Fig. 5g, the RuO_2_ catalyst exhibited a potential-dependent absorption band, corresponding to the *ν*(O–O) stretching vibration of adsorbed *OOH intermediates. The band intensity increased exponentially with applied potential from 1.4 V to 1.6 V. In contrast, Eu-RuO_2_ manifested this vibrational signature at significantly reduced overpotential (onset: 1.35 V, Fig. 5h) and displayed a stronger peak at the same potential, aligning with the decreased Gibbs free energy barrier for the O* *→* OOH* transition identified in thermodynamic profiling (Fig. 5d).^19^ Furthermore, ATR SEIRAS analysis (Fig. 5g and h) reveals that, compared with RuO_2_, Eu RuO_2_ exhibits a characteristic O–O peak near 1050 cm^−1^ at a lower applied potential, and this peak intensifies significantly with increasing potential, indicating that Eu doping promotes the OPM pathway. Furthermore, supplementary Gibbs free energy calculations confirm that the energy barrier of the rate determining step for the OPM pathway decreases from 2.013 eV to 1.855 eV upon Eu doping (Fig. S39–41). Collectively, these results demonstrate the unique advantage of Eu doping in enhancing the OPM pathway, which stands in stark contrast to the effects observed for the vast majority of other doping strategies.

## Conclusions

In summary, we successfully synthesized a novel Eu-RuO_2_ catalyst, which achieves a low overpotential of 195 mV and demonstrates remarkable durability, retaining stable activity for over 2800 h at 10 mA cm^−2^ in 0.5 M H_2_SO_4_, outperforming all previously reported Ru-based catalysts. In PEM-WE applications, the Eu-RuO_2_-based system requires only 1.67 V voltage at a current density of 1000 mA cm^−2^ and long-term stability (>300 hours at 1000 mA cm^−2^) at 60 °C in normal ambiance. Through a series of characterization studies and DFT calculations, it was revealed that the 4f buffer band of Eu donates electrons to preserve Ru–O covalency, and the Eu–O–Ru unit site suppresses the increase in the Ru oxidation state and Ru dissociation during the OER, enhancing stability. Additionally, the 4f–2p–3d gradient orbital coupling modulates adsorption interactions, boosting OER activity. This work highlights the potential of RE elements in engineering high-performance Ru-based catalysts, providing a novel strategy to overcome the challenges of PEM-WE and advance OER catalyst design for acidic environments.

## Author contributions

Yin Qin, Kaikai Li, Hao Xiang Li, and Tong-Yi Zhang managed the project and conceived the storyline of the paper. Y. Qin synthesized the materials and performed the electrochemical and structural characterization and analysis. Kang Jie Li performed the DFT calculations. Zhehan Ying contributed to the discussions of the project.

## Conflicts of interest

There are no conflicts to declare.

## Supplementary Material

SC-OLF-D6SC01665K-s001

## Data Availability

All data supporting the findings of this study are available within the paper and its supplementary information (SI). Supplementary information is available. See DOI: https://doi.org/10.1039/d6sc01665k.
