# Supplementary material for: Enhancing acidic oxygen evolution reaction with a highly stable Eu-doped RuO2 electrocatalyst
Source: Chem Sci. 2026 Aug 7;17(37):17980–9. doi: 10.1039/d6sc01665k (PMC13448965; doi:10.1039/d6sc01665k)
Supplement: Supplementary file 1 [file SC-017-D6SC01665K-s001.pdf]

Supplementary Information (SI) for Chemical Science.
This journal is © The Royal Society of Chemistry 2026

Supporting Information (SI)

Engineering gradient 4*f*-2*p*-3*d* orbital coupling

According to the symmetry-adapted linear combination (SALC) theory, the 4*f* orbitals of rare-earth (RE) elements can hybridize with O 2*p* orbitals through σ- and π-type bonding interactions—an effect that becomes particularly significant under the local O_h symmetry of the [TMO_6] octahedral unit. [1, 2] On the basis of this principle, we have developed rare-earth-doped RuO_2 materials to improve their catalytic properties, and we examined a range of doping configurations, from which the optimal one was eventually identified. In transition-metal oxide systems, the covalency between M and O ions gives rise to both occupied bonding (M–O) and vacant antibonding (M–O*) *bands, and the electron occupation dynamics within these bands are decisive for the material's electrical conductivity. Under the Mott–Hubbard description, the strong on-site Coulomb repulsion (U), originating from d–d electron correlations, drives a symmetry-breaking split of the antibonding (M–O*)* band into a filled lower Hubbard band (LHB) and an empty upper Hubbard band (UHB). The energy separation between the bonding and antibonding band manifolds, commonly referred to as the charge-transfer energy (Δ), is dictated by both the electronegativity difference between M and O and the Madelung potential imposed by the lattice structure.[21 3] Crucially, U exhibits an inverse dependence on the spatial spread of d orbitals and the cationic oxidation state. At the same time, the combined influence of charge-transfer and Mott–

Hubbard physics governs the hybridization strength of 3d electrons in the vicinity of the Fermi level (EF). The antagonistic interplay between U and Δ generates an adjustable electronic framework, thereby serving as a mechanistic handle to tailor the nature of M–O bonds via selective tuning of orbital overlap and electronic correlation.[21]

Under the Mott–Hubbard splitting framework, when the charge-transfer energy Δ dominates over the on-site Coulomb and exchange interactions (i.e., $U \ll \Delta$), the anodic polarization during OER drives electron removal from the lowest occupied band—specifically, the LHB associated with the M–O manifold. This process triggers excessive oxidation, which not only weakens the M–O covalency but also yields both deactivated and highly reactive O^{n-} species. To counteract this degradation, the RE(f)–O(p)–TM(d) unit is deliberately constructed, wherein the f-orbital level is positioned slightly above the LHB to serve as a buffer reservoir. By preserving the occupancy of the LHB, these buffering states effectively safeguard the integrity of TM–O bonding. The electronic buffering mechanism arises from: (i) The ionic nature of the RE–O bond, which facilitates electron donation from RE to O due to the strong RE–O affinity; (ii) The intrinsically elevated energy of *f*-orbitals and the low ionization energy of RE species, positioning the *f*-band closer to E_F. By maintaining the LHB through gradient orbital coupling in the TM–O–RE unit, this design suppresses M–O covalency loss and overoxidation, thereby offering stable and efficient active sites for OER.[21]

Experimental Sections.

Chemicals and materials. Ruthenium chloride hydrate ($RuCl_3 \cdot xH_2O$, 35.0-42.0% Ru basis), Europium standard solution (1000 ug/ml in 10% HCl), Cerium(III) nitrate hexahydrate ($Ce(NO_3)_3 \cdot 6H_2O$), Samarium(III) nitrate hexahydrate ($Sm(NO_3)_3 \cdot 6H_2O$), Iridium dioxide (IrO_2), Ruthenium oxide (RuO_2), carbon nanotubes (CNT), and 5 wt.% Nafion® solution were all sourced from Aladdin Reagent (Shanghai) Co., Ltd. $NH_3 \cdot H_2O$ was obtained from Macklin Co., Ltd. Sulfuric acid (H_2SO_4) solutions and ethanol (C_2H_5OH) were procured from Sinopharm Co., Ltd. All chemicals used were of analytical grade and were used as received without further purification. The de-ionized water with a resistivity of 18 MΩ cm^{-1} was purified using an ultra-pure purification apparatus (Aqua Solutions) to ensure sanitization.

Synthesis of Eu-RuO_2 and RuO_2: Eu-doped RuO_2 was synthesized through a hydrothermal method. First, 30 mg of analytical-grade $RuCl_3 \cdot xH_2O$ was dissolved in 1 mL of deionized water to prepare a homogeneous $RuCl_3$ solution. Next, 600 μL of this solution and a certain amount of Eu standard solution (or equivalent molar amounts of Ce and Sm nitrates) were added to 10 mL of deionized water in a 20 mL glass bottle. The mixture was stirred for 15 minutes and then transferred to a Teflon-lined vessel containing some ammonia solution. The vessel was sealed, placed in an autoclave, and heated at 140°C for 6 hours. After the reaction, the precipitate was collected, washed twice with ethanol, and dried. The dried product was then calcined in a muffle furnace

at 300 °C for 6 hours in air to yield Eu-RuO_2. For comparison purposes, pure RuO_2 was prepared without the addition of the Eu standard solution.

Characterization: TEM images were acquired using the Thermo Fisher Scientific Talos F200X microscope at 200 kV, and elemental composition and distribution were analyzed via EDS element mappings. SEM images were captured via the Regulus8100 microscope, which had a 2 kV operating voltage. XRD patterns of powder samples were measured on the Malvern PANalytical AERIS X - ray powder diffractometer using Cu Kα radiation (λ = 0.154 nm). XPS measurements were performed on the ULVAC-PHI PHI VersaProbe4 spectrometer equipped with an Al Kα monochromatic X-ray source, with all binding energies calibrated to the C 1*s* peak at 284.8 eV. XAS spectra at the K-edge of Ru were collected in transmission mode at the BL14W1 beamline of the 9-50 KeV synchrotron radiation source at SSRF, China. Raman spectra were obtained on the Renishaw inVia using a 532 nm excitation laser.

Electrochemical measurements: Electrochemical tests for Com-RuO_2, RuO_2, and Eu-RuO_2 were carried out in 0.5 M H_2SO_4 electrolyte at room temperature using a standard three-electrode setup controlled by an electrochemical workstation. Before the tests, oxygen was bubbled through the 0.5 M H_2SO_4 electrolyte for 10 minutes to ensure oxygen saturation. The working, reference, and counter electrodes were a catalyst-coated glassy carbon electrode (5 mm diameter), an Ag/AgCl electrode, and a carbon rod, respectively.

In a typical preparation, 4 mg of catalyst powder was added to a premixed solution of 200 μL ethanol and 200 μL Nafion aqueous solution (0.5 vol.%, with ethanol as solvent). The mixture was ultrasonicated for 15 minutes to form a uniform black ink. Then, 5 μL of the ink was drop-cast onto a glassy carbon (GC) electrode, yielding a Ru loading of approximately 0.536 mg cm^{-2}. Linear sweep voltammetry (LSV) curves were obtained using a voltage range of 1.0 V to 1.6 V vs. RHE, with a scan rate of 5 mV s^{-1}; iR-compensation was not applied. Cyclic voltammetry (CV) measurements were performed in the non-Faradaic region at scan rates of 5, 10, 20, 30, 40, and 50 mV s^{-1}. A symmetric potential window centered about the open-circuit potential was selected, ensuring that only charging of the electrode/electrolyte interface occurs within this window, free from any Faradaic current interference. The electrochemically active surface areas (ECSA) were estimated from the electrochemical double-layer capacitance (C_{dl}) of the catalytic surface. C_{dl} was determined by plotting $\Delta J/2$ ($\Delta J = J_a - J_c$, where J_a is the anodic current density and J_c is the cathodic current density at the middle voltage) against the scan rate, with the slope representing C_{dl}. Using a specific capacitance (C_s) of 0.035 mF cm^{-2}, [4]

ECSA was calculated as ECSA = $C_{dl} \cdot S/C_s$, and S corresponds to the geometric surface area of the working electrode. For the stability test, a carbon cloth (CC) coated with the catalyst was employed as the working electrode, which was prepared by drop - casting 400 μL of the catalyst ink onto a 1 cm × 1 cm CC. The turnover frequency (TOF) values were obtained by using the following equation: [5, 6] TOF = $(J \times A \times \zeta)/(4 \times F \times n)$ where J is the current density at the overpotential of 230 mV, A is the electrode

geometric area, ζ is the faradaic efficiency and equals 1 in present case, F is the Faraday constant, and n is the mole number of Ru sites based on all the Ru atoms. Chronopotentiometric measurements were carried out at a constant current density of 10 mA cm^{-2} using carbon cloth as the substrate, and at 100 mA cm^{-2} using carbon paper.

Operando DEMS with O isotope labelling: *Operando* DEMS with isotope O labeling tests were carried out on a QAS 100 quadrupole mass spectrometer (Linglu Instruments, Shanghai) attached to an electrolytic bath with a traditional three-electrode system. The preparation method for the electrocatalyst ink is described in the section on electrochemical measurements. Specifically, 100 μl of this ink was drop-cast onto an Au mesh working electrode with an area of 1.0 cm^2 and allowed to dry under ultraviolet light. The electrolytic bath was initially subjected to ^{18}O labeling in a 0.5 M H_2SO_4 solution made with ^{18}O-enriched water (97 atom % ^{18}O). This process was carried out using cyclic voltammetry (CV) with a potential range of 1.00 to 1.55 V vs. RHE at a scan rate of 5 mV s^{-1}. Following the initial labeling, all electrodes and electrolytic cells were thoroughly rinsed with ^{16}O-labeled water to eliminate any residual $H_2{}^{18}O$. Subsequently, the system was operated in a 0.5 M H_2SO_4 solution containing $H_2{}^{16}O$, and cyclic voltammetry (CV) was performed at a scanning rate of 5 mV s^{-1} over the potential range of 1.1 to 1.55 V vs. RHE to detect mass-selected products with m/z ratios of 32, 34, and 36.

Operando XAS: Operando XAS measurements were conducted in fluorescence mode at the BL14W1 beamline of the Shanghai Synchrotron Radiation Facility (SSRF),

utilizing a 9-50 keV synchrotron radiation source. The working electrode was prepared by depositing the catalyst onto carbon paper with a loading density of 4 mg cm^{-2}. A three-electrode configuration was employed, with a carbon rod serving as the counter electrode and an Ag/AgCl electrode functioning as the reference electrode. The electrode assembly was integrated into an organic glass electrochemical cell containing 0.5 M H_2SO_4 electrolyte. Spectroscopic data acquisition was performed under applied oxidation potentials of 0, 1.3, 1.4, 1.5, and 1.6 V (vs. RHE), with Ru foil serving as reference standards. Experimental data processing and EXAFS fitting were systematically executed using the Athena and Artemis software packages. [7]

In situ ATR-SEIRAS measurements: In situ ATR-SEIRAS measurements were performed using a Nicolet 6700 Fourier-transform infrared spectrometer equipped with an EC-IR-II at a fixed incident angle. The system was configured with a spectral resolution of 4 cm^{-1}, employing cumulative averaging of 32 interferograms per spectrum to enhance signal-to-noise ratio. The working electrode was fabricated through a two-step procedure: (i) an ~50 nm Au film was chemically deposited onto a hemispherical ZnSe prism via electroless plating to facilitate plasmon-enhanced infrared signal detection; (ii) subsequently, the catalyst ink (0.1 mg cm^{-2} loading) was precisely drop-cast onto the Au-modified substrate. This electrode assembly was integrated into a custom-designed spectro-electrochemical cell with a three-electrode configuration, where a Pt coil and Ag/AgCl electrode served as counter and reference electrodes, respectively. The electrochemical interface was maintained using high-purity 0.5 M H_2SO_4 electrolyte that had been rigorously deoxygenated by Ar purging.

All potential-dependent SEIRAS spectra were acquired synchronously with potentiostatic measurements within the potential range of 1.0-1.6 V vs. RHE.

PEM water electrolyzer tests: In the PEM-WE setup, the anode catalyst was either synthesized RuO_2 or Eu-RuO_2 loaded at ~2.103 mg cm^{-2} (the Ru loading is approximately 1.598 mg cm^{-2}, corresponding to a cost of about 0.021 USD), while the cathode used commercial Pt/C (40 wt.% Pt) at ~0.5 mg cm^{-2}. A Nafion 115 membrane acted as the PEM, which was pretreated by soaking in 5% H_2O_2 at 80 °C for 30 min, then rinsing with 0.5 M H_2SO_4 and ultrapure water to eliminate contaminants. Iridium-coated titanium fiber felt and carbon paper served as anode and cathode GDLs, respectively. The Eu-RuO_2 sample was ultrasonically treated in ethanol, settled, with the upper layer collected to get smaller particles. The anode and cathode catalyst inks were made via microwave-assisted ultrasonication, mixing the catalyst (Eu-RuO_2 for the anode; Pt/C for the cathode) with 5% Nafion, isopropanol, and deionized water. The anode ink followed a weight ratio of 2:6:70:10 (Catalyst: Nafion: isopropanol:water), and the cathode ink a ratio of 2:10:80:10. Both were sonicated for 1 h for uniform dispersion. The catalyst ink was evenly applied, half sprayed onto the PEM and half onto the GDL surfaces. The catalyst-impregnated PEM and GDLs were hot-pressed at 120 °C for 3 minutes under 0.3 MPa to form the MEA, which was then enclosed between two titanium bipolar plates with serpentine flow channels (1 cm × 1 cm reaction area) to assemble the PEM-WE cell. The PEM-WEs were tested at 60 °C using distilled water as the electrolyte, pumped through the anode at 8 mL min^{-1} by a peristaltic pump (model BT100-3J). Polarization curves were recorded across a current

density spectrum spanning 0 to 2500 mA cm^{-2}. Stability characterization utilized chronopotentiometry at target current densities of 500 and 1000 mA cm^{-2}, enabling durability assessment under relevant operational conditions.

DFT calculations: The DFT calculations were performed using the Vienna ab initio Simulation Package (VASP) [8, 9]. Perdew, Burke, and Ernzerhof (PBE) functional of generalized gradient approximation (GGA)[10] with projector augmented wave (PAW)[11] was applied to describe the electronic structures of materials. The plane-wave basis kinetic energy cut-off was set to 500 eV. For the structural optimization of RuO_2(110)-slab, The Brillouin-zones are sampled using Monkhorst-Pack k-mesh of 3×2×1. For the calculations of the OER process, Van der Waals interaction is considered using the D3 method. The vacuum layers are set to ~15 Å to decouple the interaction between periodic images. The Brillouin-zones are sampled using Gamma centered k-mesh of 3×2×1. The RuO_2(110) surface was modeled by a 2 × 4 × 2 supercell (with 90 O atoms and 48 Ru atoms), in which the top two atomic layers are allowed to relax, and the bottom two layers are fixed. As for Eu-doping-RuO_2 (110), two Ru atoms in the RuO_2(110) model were substituted by Eu atoms. The geometry relaxation and convergence criteria for the electronic structure were 0.02 eV $Å^{-1}$ and 1×10^{-5} eV, respectively. In the DFT calculations for Eu^{2+} and Eu^{3+} doping into RuO_2, distinct Eu pseudopotential files were adopted: specifically, the Eu_2 pseudopotential was used for Eu^{2+}, while the Eu_3 pseudopotential was applied for Eu^{3+}. The gas-phase H_2 and H_2O molecules are optimized in a box of dimensions 15 Å×15 Å×15 Å with Gamma point sampling of the Brillouin-zone. The adsorption energy (E_{ad}) is calculated by

$$E_{ad} = E_{total} - E_{slab} - E_{adsorbate},$$

where E_{total} refers to the total energy of the optimized structure with the adsorbates absorbed on the slab surface, E_{slab} refers to the energy of the clean slab, and $E_{adsorbate}$ refers to the energy of the adsorbate (O*, OH*, and OOH*) in vacuum. The free energy of the reaction steps were calculated by the equation: $\Delta G_{ads} = \Delta E_{ads} + \Delta E_{ZPE} - T\Delta S$, where ΔE_{ads} is the adsorption energy of intermediates, and T is temperature, which is 298.15K. ΔE_{ZPE} and ΔS represent the energy variance in zero-point energy and entropy, respectively. As for the dissolution energy of Ru atom, the following equation is used: $\Delta E_{VRu} = E(Ru_V) + E(Ru) - E(initial)$, where $E(Ru_V)$ is the total energy of Ru vacancy surface, $E(Ru)$ is the energy of Ru bulk, and $E(initial)$ is the total energy of initial surface before demetallation of Ru. The process of calculating the dissolution energy of O atom is similar to that of Ru atom.

Supplemental Fig.s and Tables.

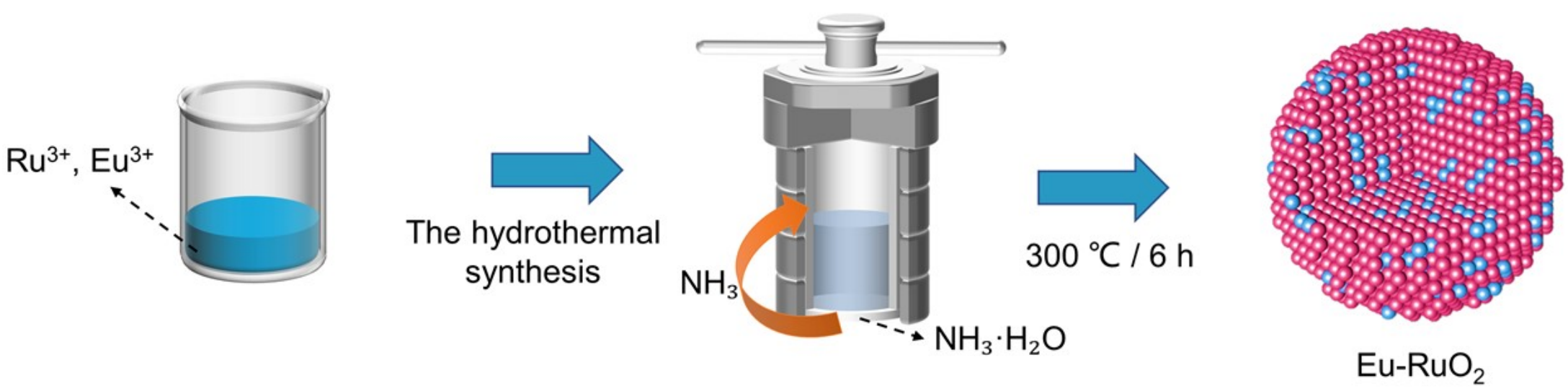

Fig. S1. Synthesis schematic diagram of Eu-RuO_2.

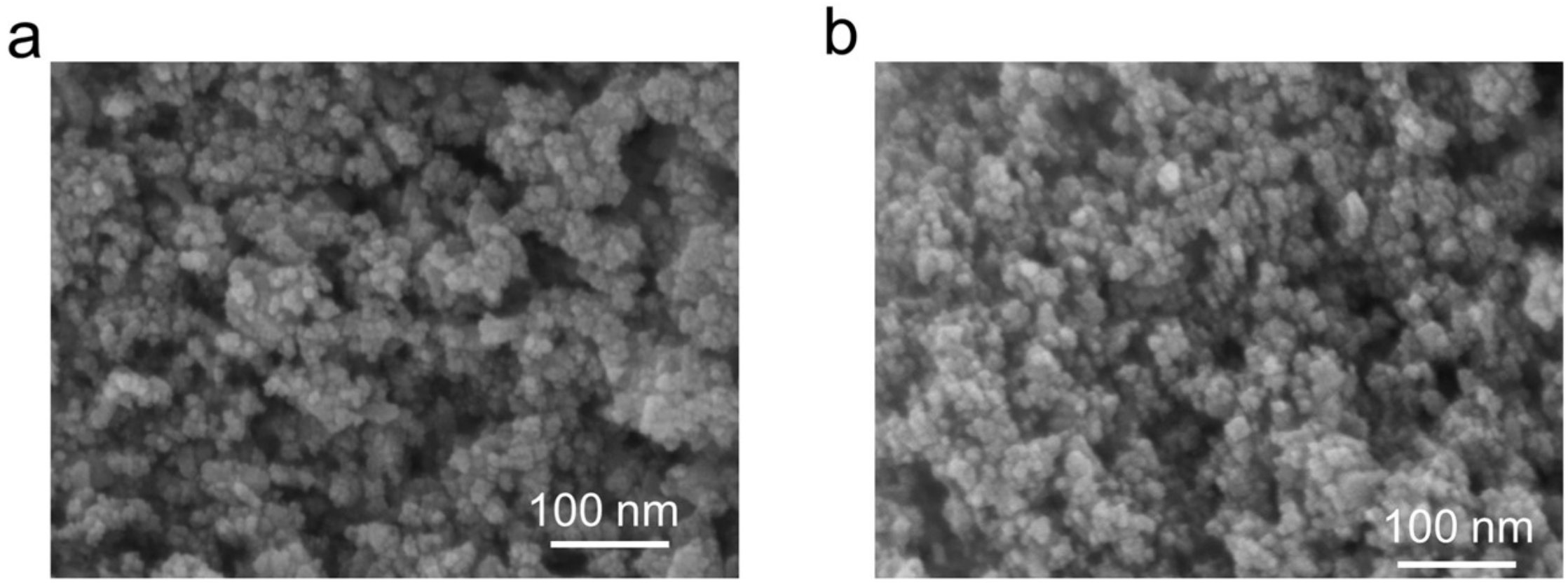

Fig. S2. SEM images of RuO_2 and Eu-RuO_2.

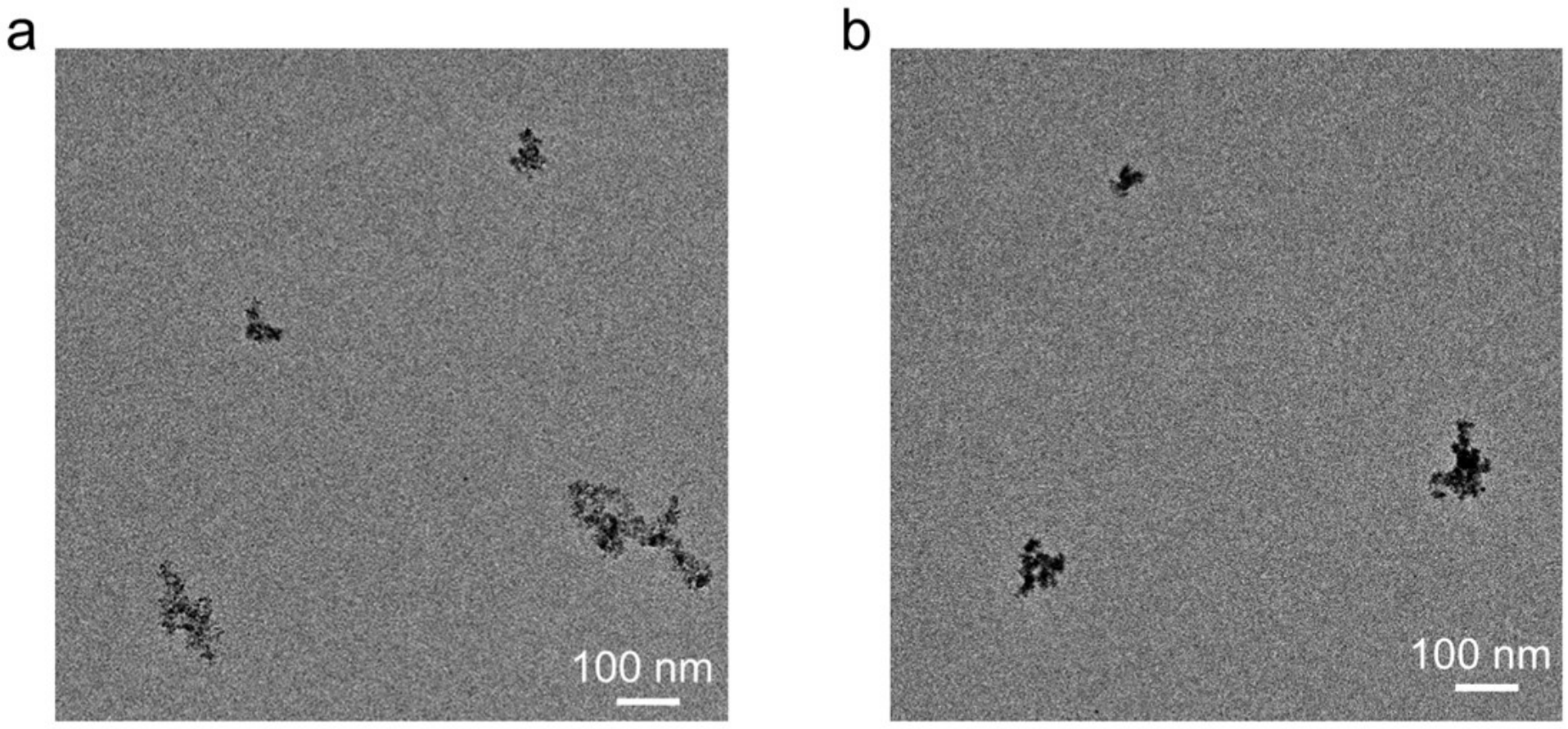

Fig. S3. TEM images of RuO_2 and Eu-RuO_2.

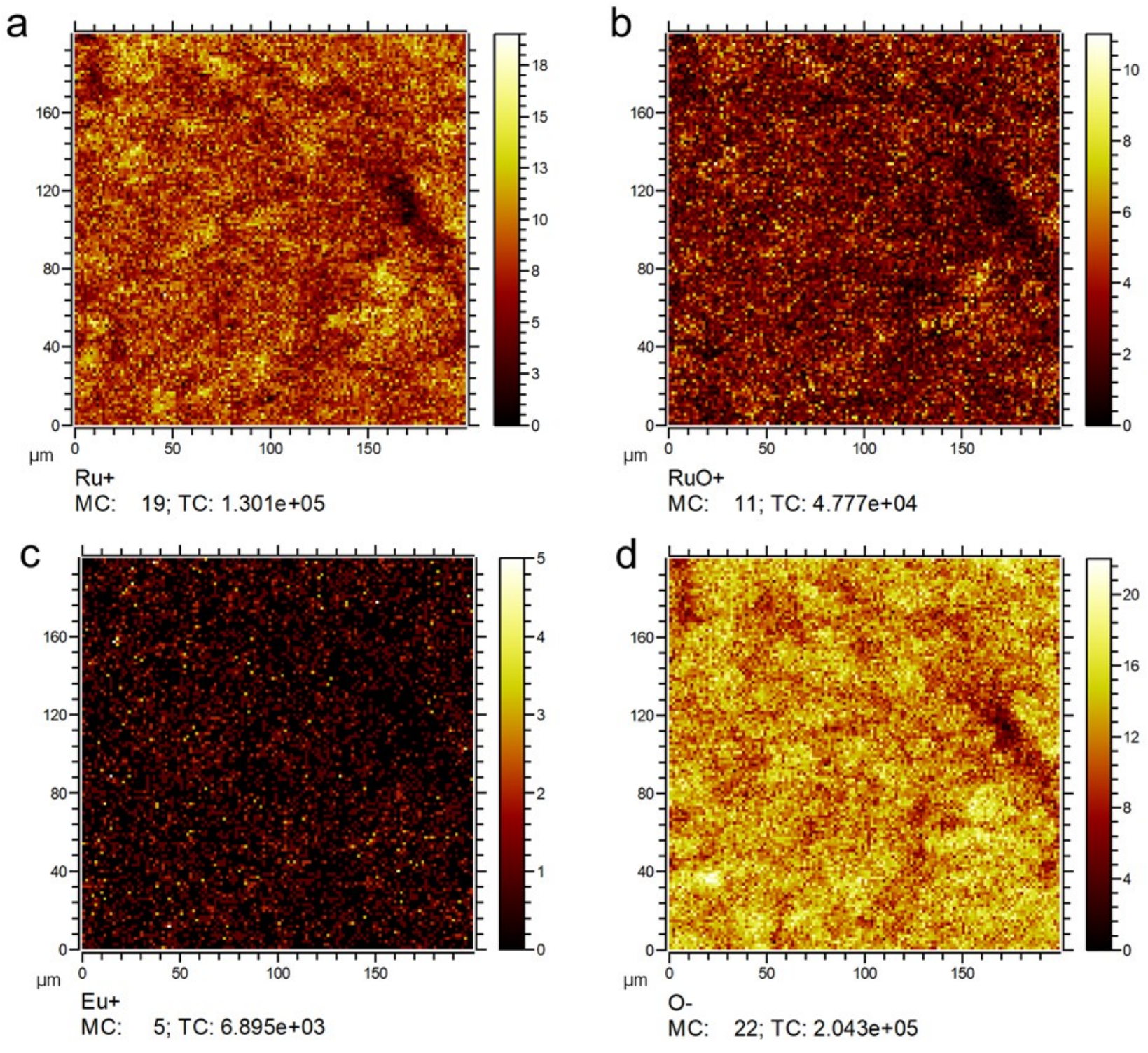

Fig. S4. TOF-SIMS images of Eu-RuO_2.

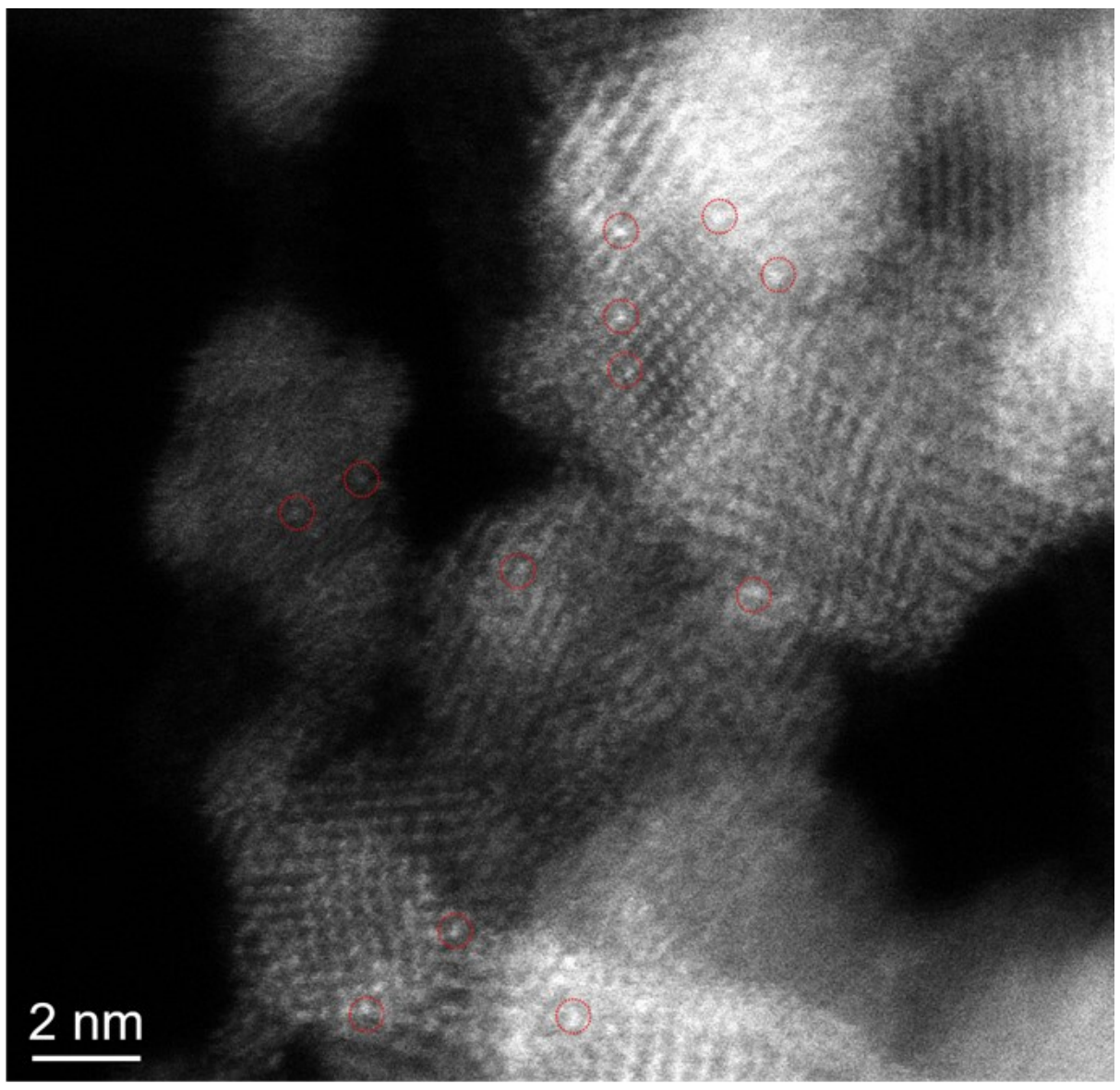

Fig. S5. HAADF-STEM image showing the Eu SAs in the RuO_2 lattice, and the inset is its enlarged view.

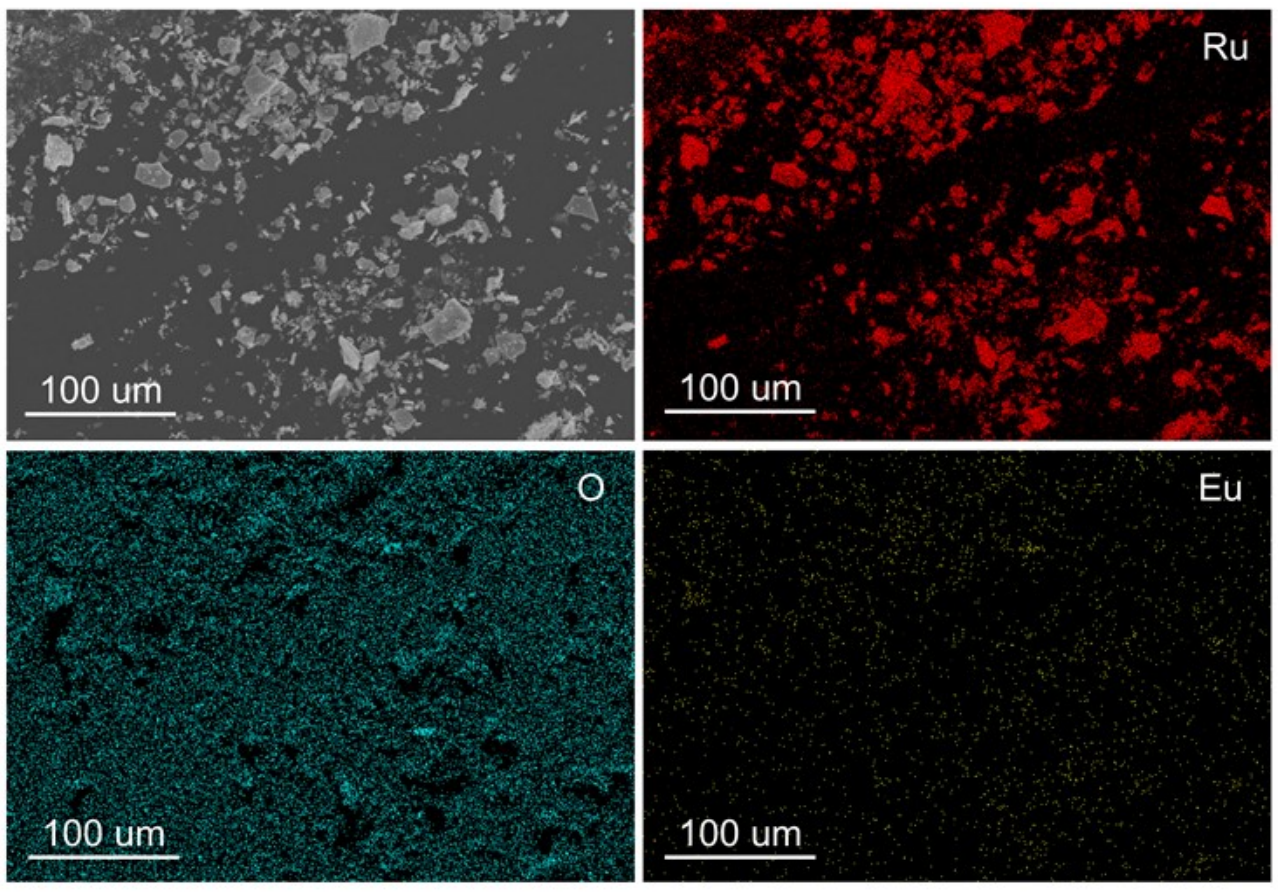

Fig. S6. SEM and corresponding elemental mapping images of Ru, Eu, and O for the Eu-RuO_2 sample after immersion in 0.5 M HCl for approximately 48 h.

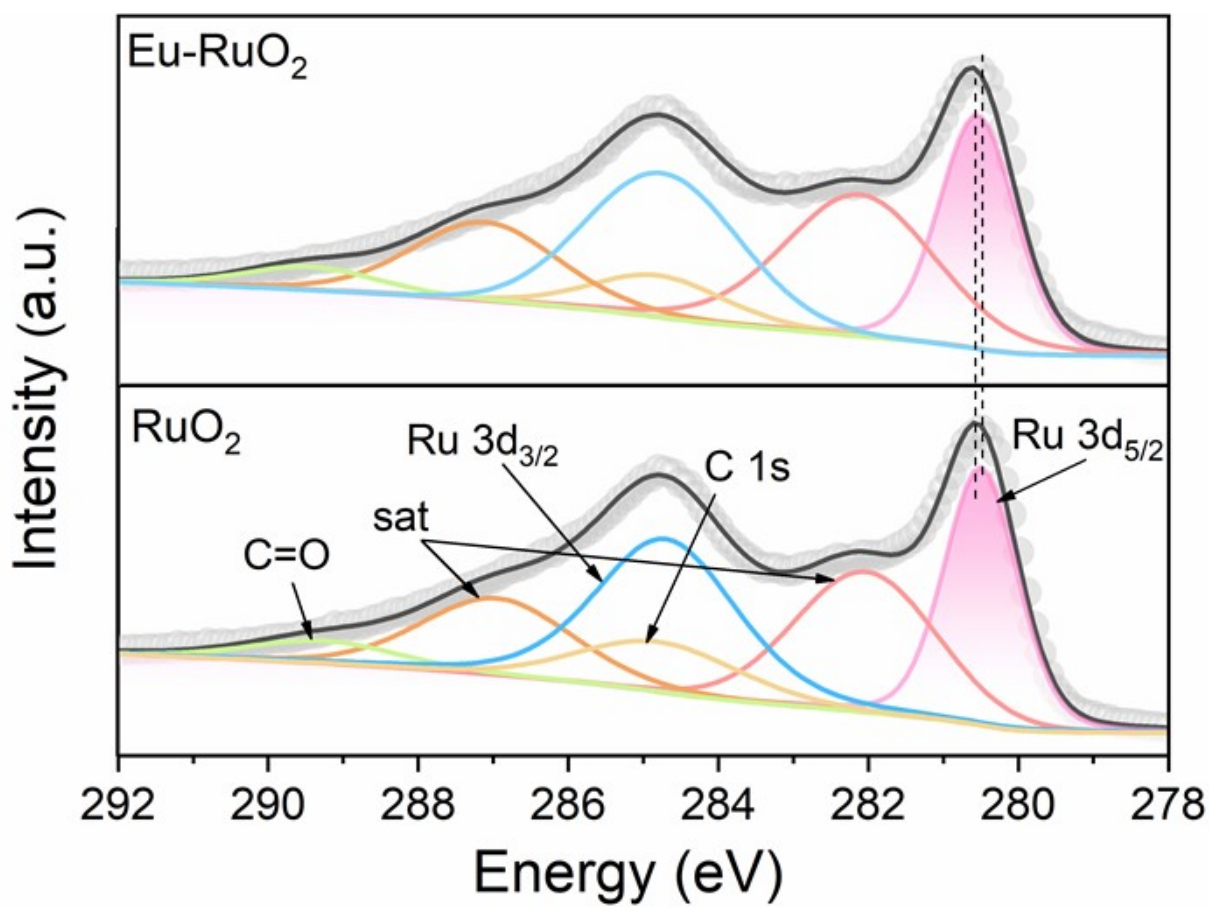

Fig. S7. The high-resolution Ru 3*d* XPS of RuO_2 and Eu-RuO_2.

For both the RuO_2 and Eu-RuO_2 samples, the full width at half maximum (FWHM) of the Ru $3d_3/_2$ peak measures 2.21 eV and 2.40 eV, respectively, corresponding to approximately 1.9–2.0 times that of the Ru $3d_5/_2$ peak (1.15 eV and 1.20 eV, respectively). The spin–orbit splitting (SOS) between the two peaks is consistently 4.2 eV for both samples. Furthermore, the peak area ratios of Ru $3d_3/_2$ to Ru $3d_5/_2$ are determined to be approximately 1.65 for RuO_2 and 1.67 for Eu-RuO_2, which are in close agreement with the theoretical value of 1.5.

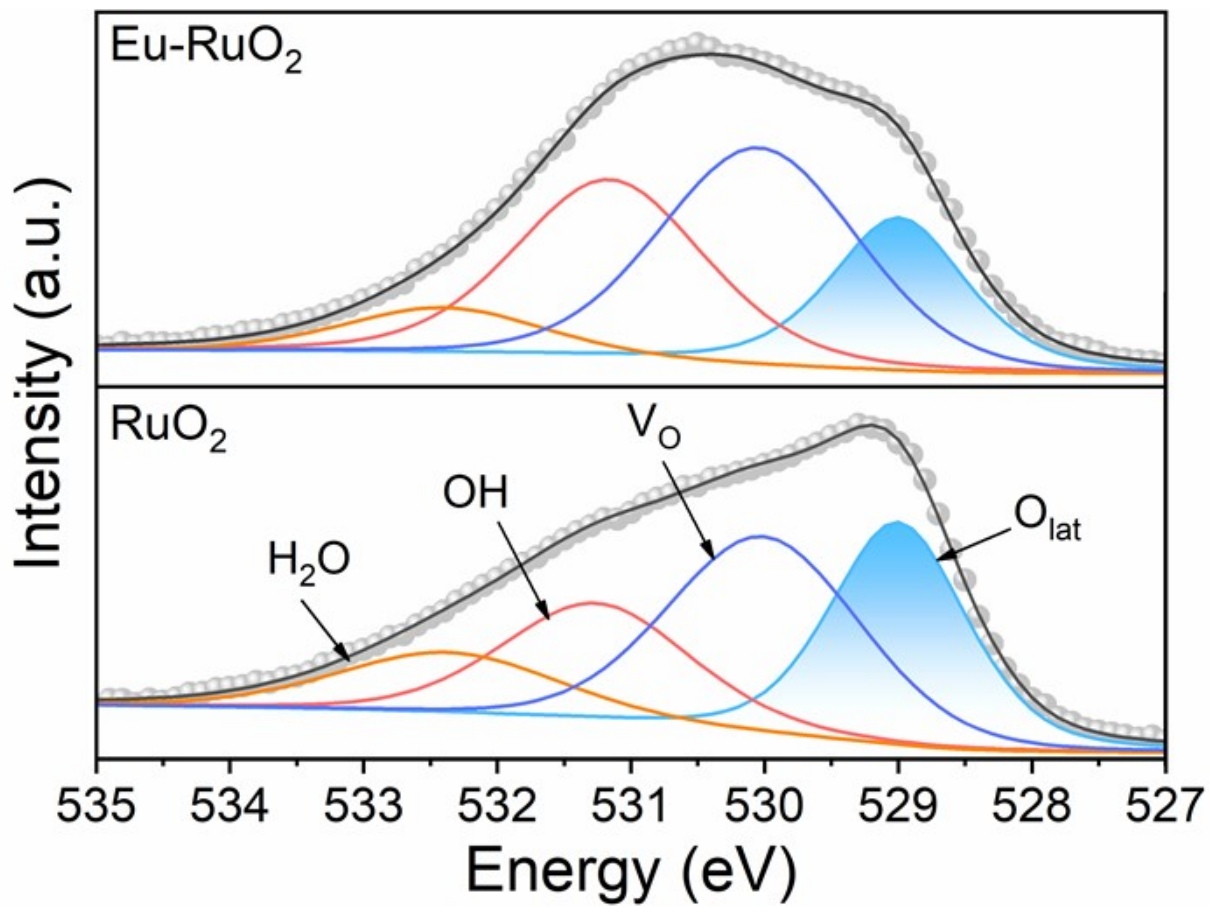

Fig. S8. The high-resolution O 1*s* XPS of RuO_2 and Eu-RuO_2.

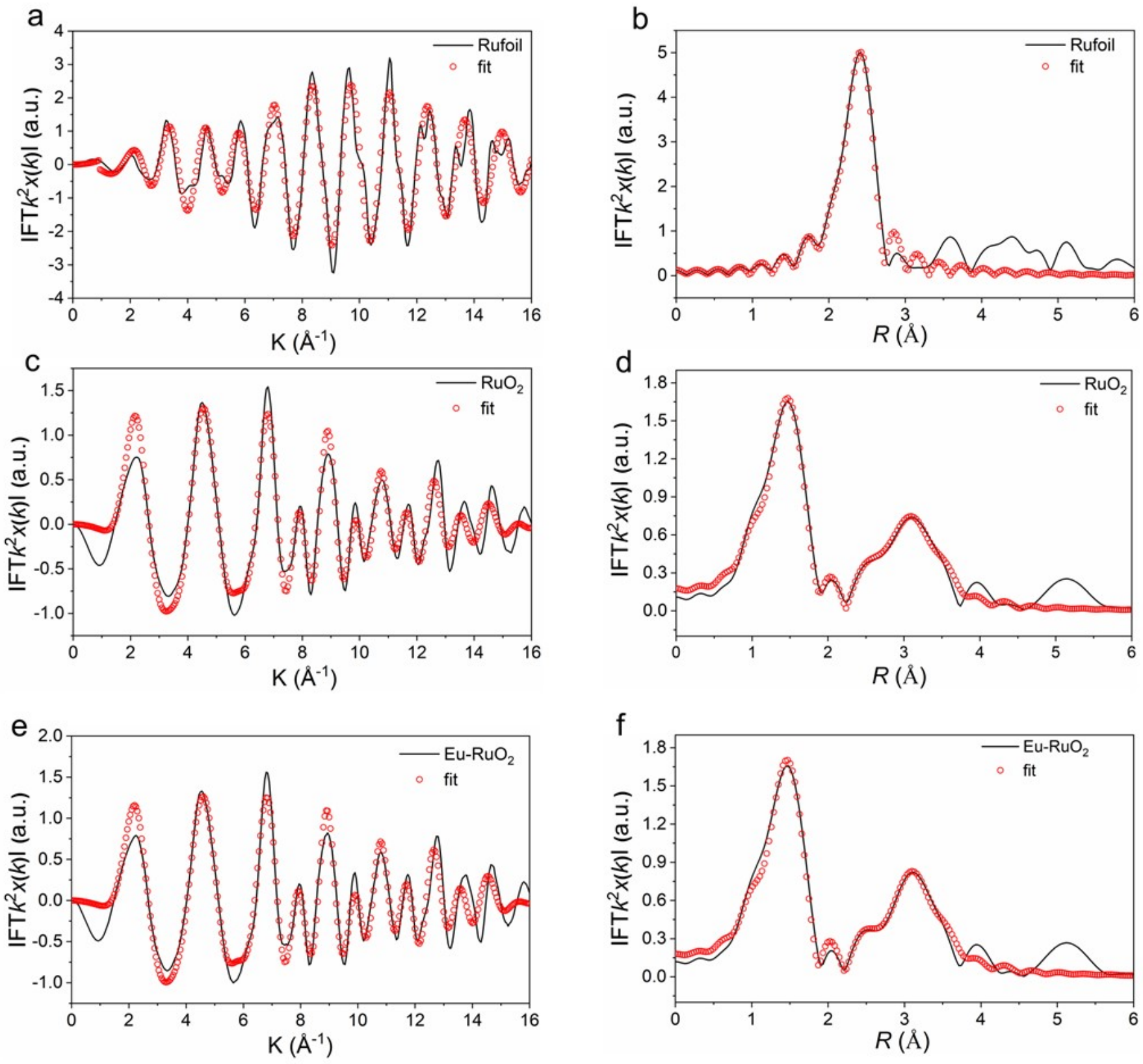

Fig. S9. The Fourier-transformed fitting Ru K-edge EXAFS spectra of Ru foil (a, b), RuO_2(c, d), and Eu-RuO_2 (e, f).

Table S1. EXAFS fitting parameters at the Ru K-edge for various samples（S_0^2=0.79）

	shell	CN	R(Å)	σ^2
Ru foil	Ru-Ru	12*	2.67±0.01	0.0036
RuO_2	Ru-O	6.8±0.3	1.47±0.01	0.0041
	Ru-Ru	11.2±1.3	3.16±0.01	0.0125
Eu-RuO_2	Ru-O	6.3±0.3	1.47±0.01	0.0033
	Ru-Ru	10.0±1.2	3.15±0.01	0.0120

[a]*CN*: coordination numbers; [b]*R*: bond distance; [c]σ^2: Debye-Waller factors;

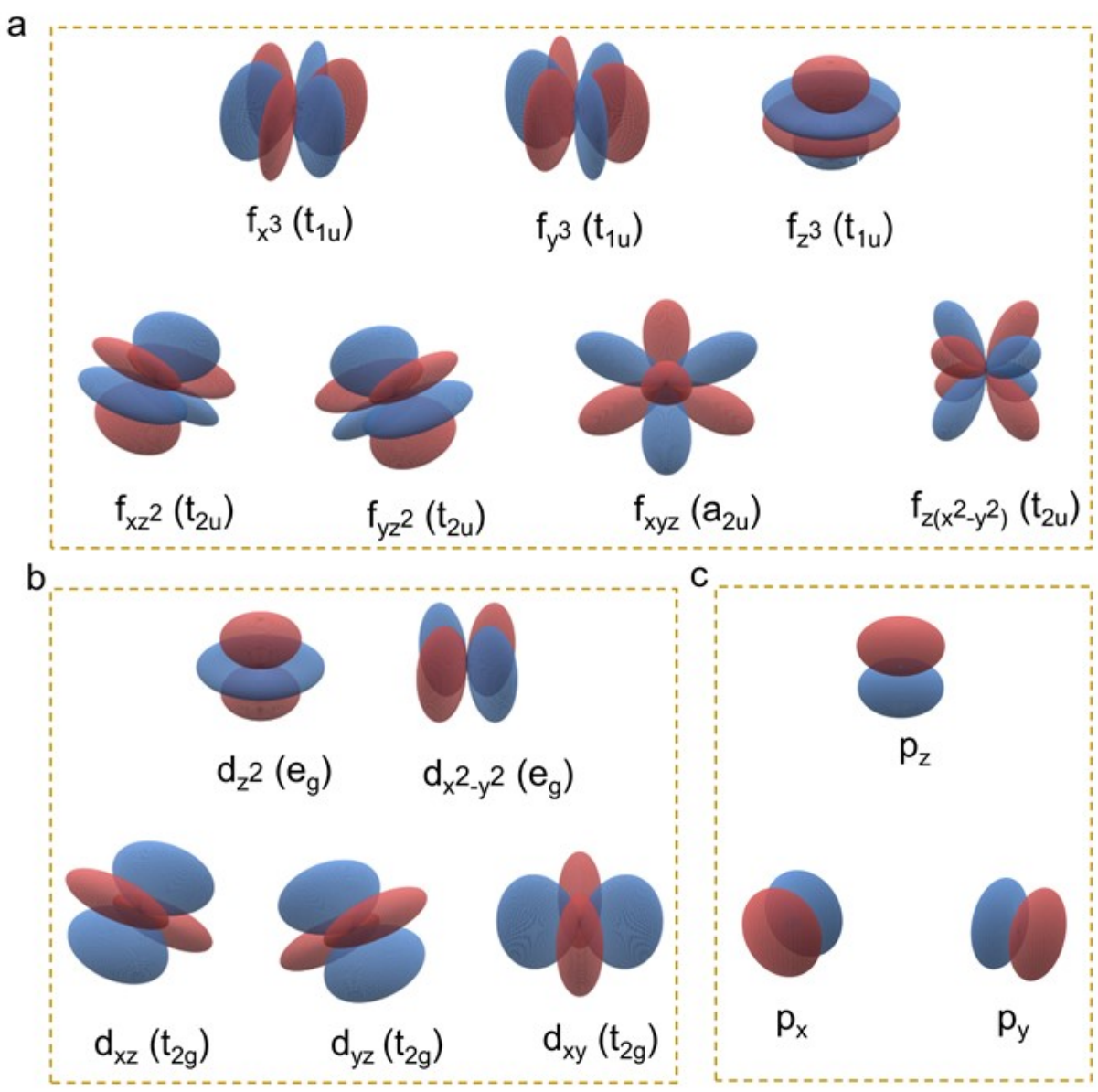

Fig. S10. (a) *d* orbital diagram; (b) *f* orbital diagram; (c) *p* orbital diagram of O.

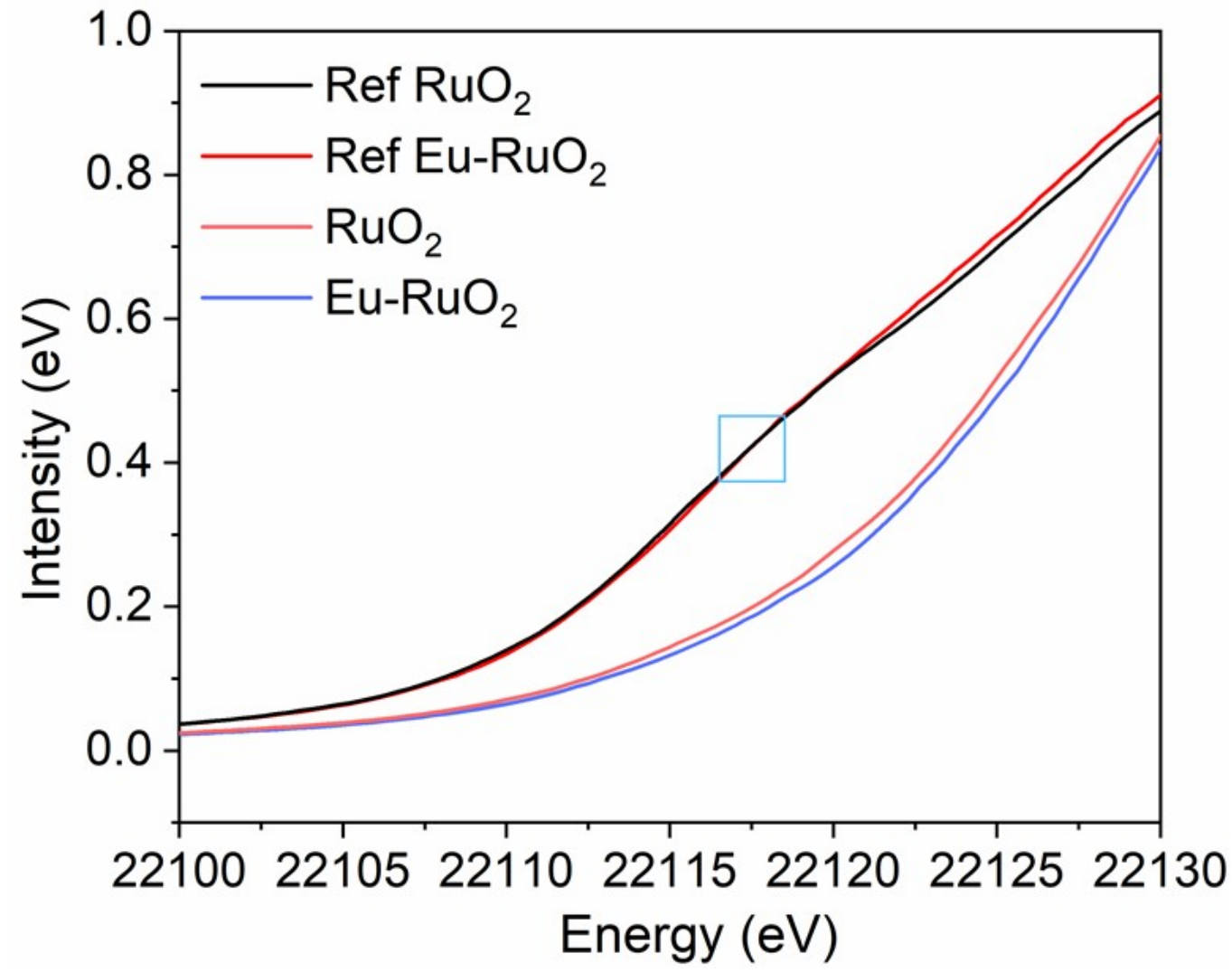

Fig. S11. Ru K-edge XANES spectra of Ru foil, RuO_2, and Eu-RuO_2.

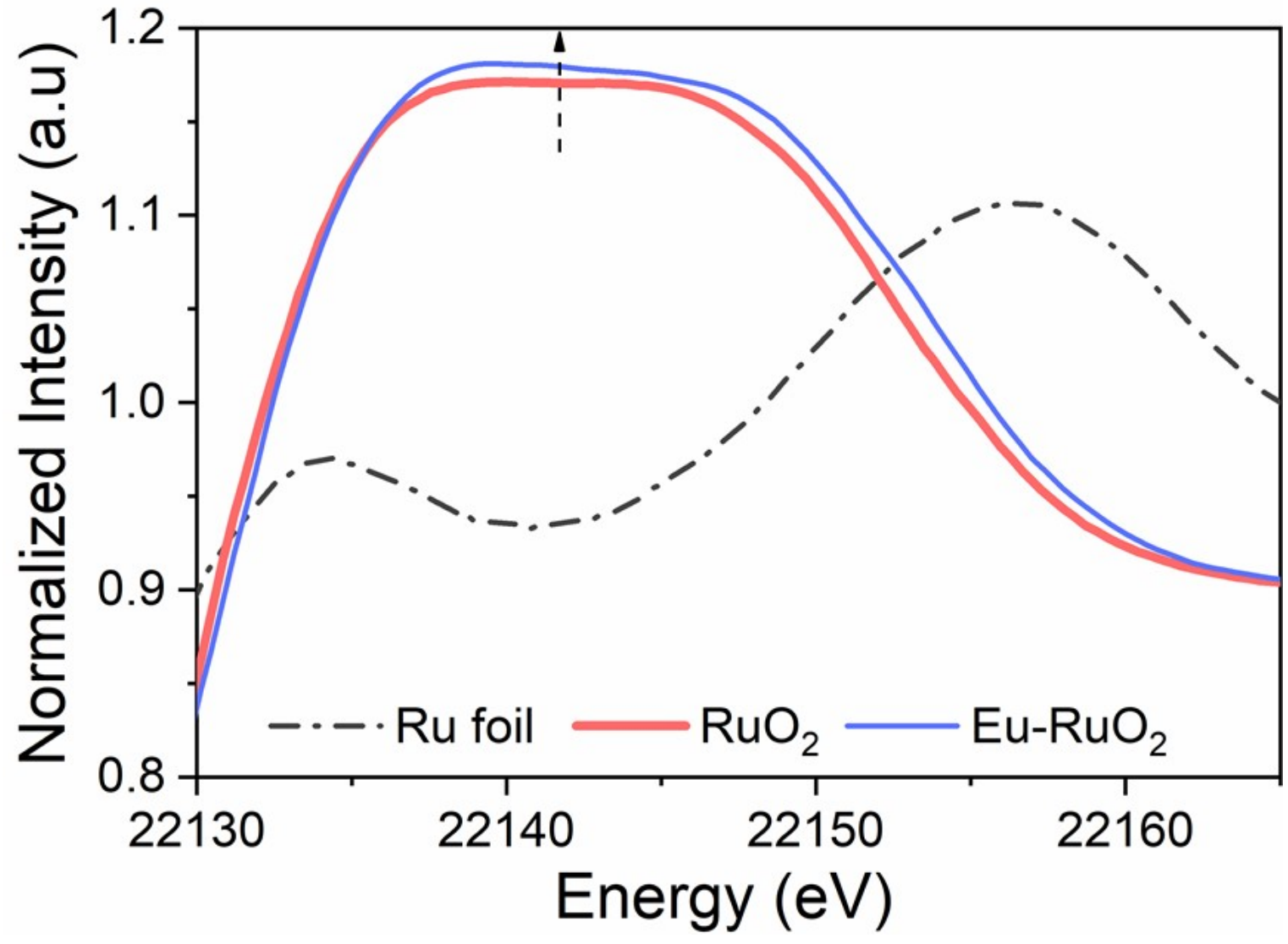

Fig. S12. Ru K-edge XANES spectra of Ru foil, RuO_2, and Eu-RuO_2.

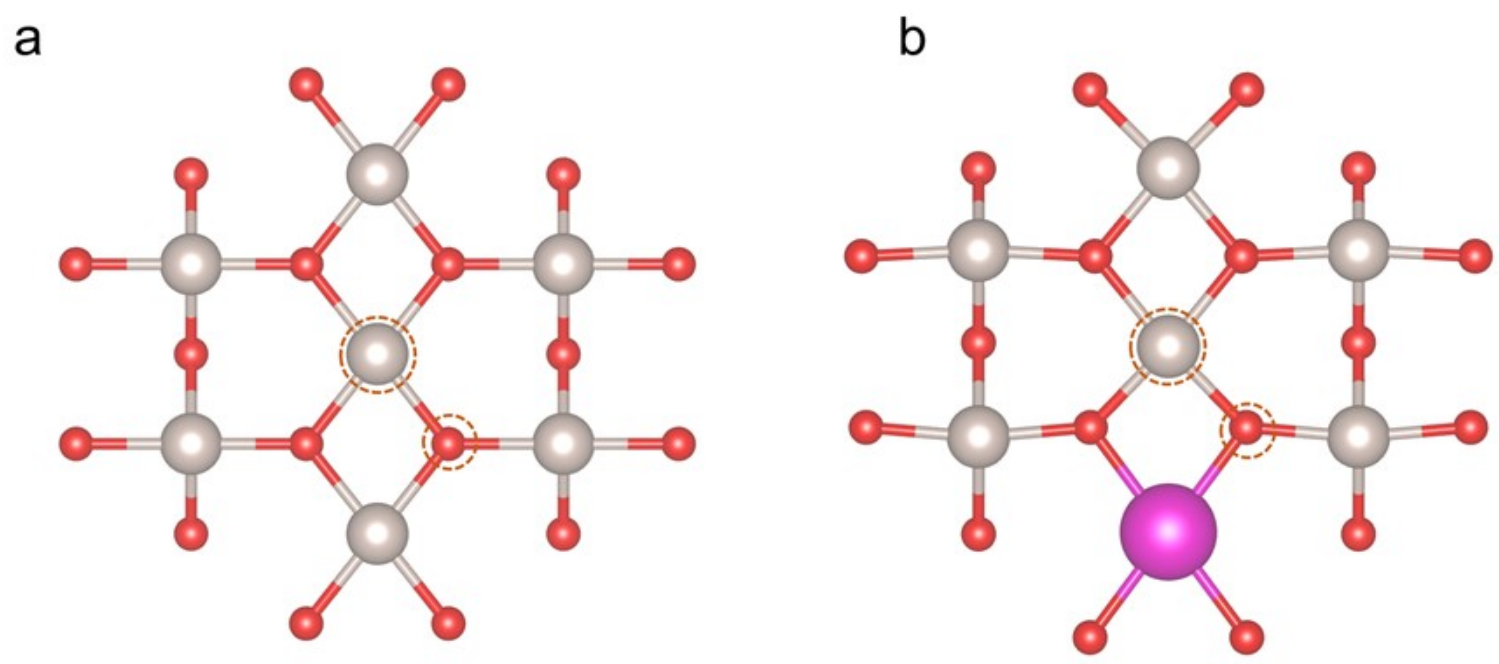

Fig. S13. Calculated DFT sites for Ru and O Bader charge.

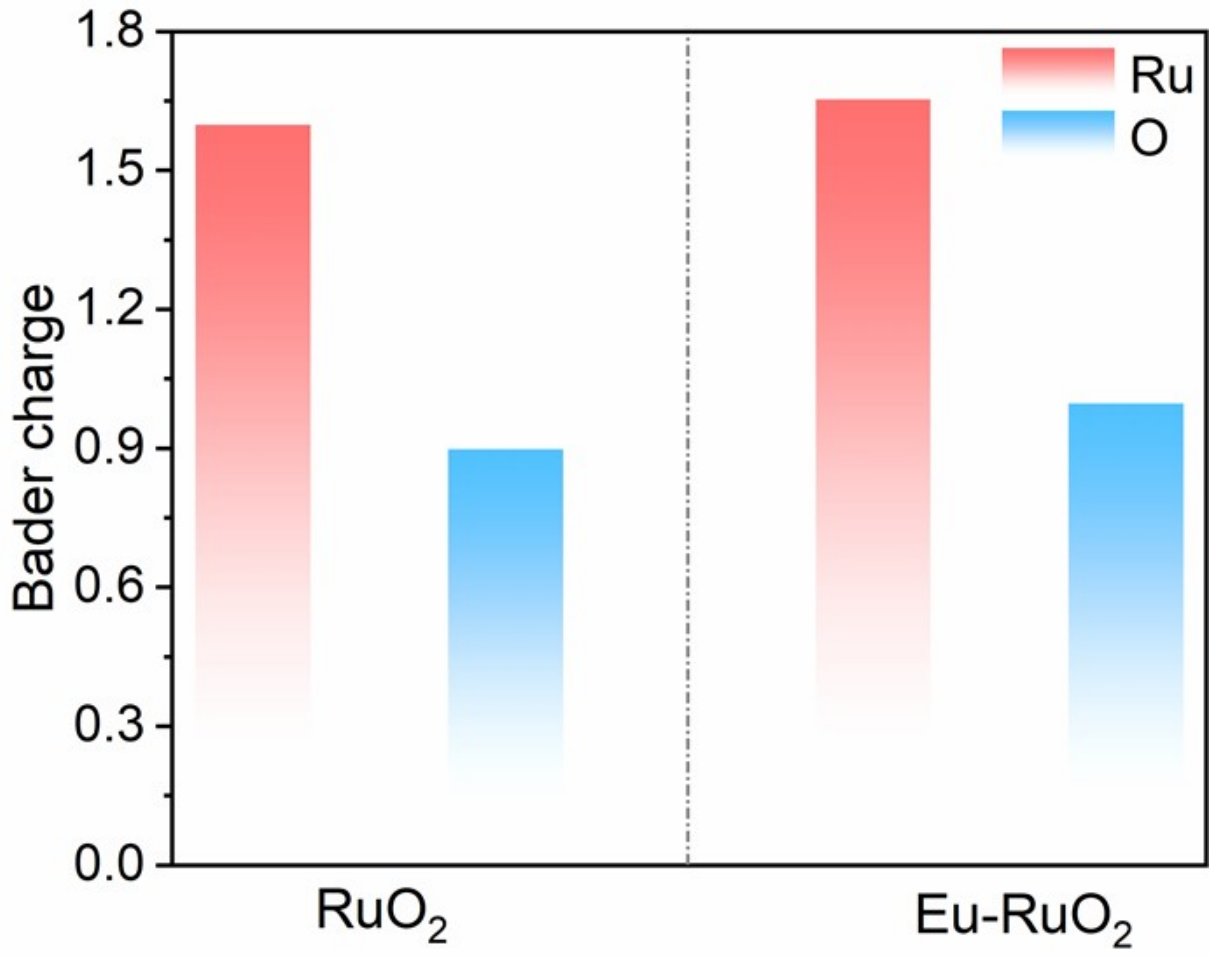

Fig. S14. Bader charge of Ru and O in RuO_2 and Eu-RuO_2.

The results reveal that O gains more electrons while Ru loses more electrons, collectively elucidating the pronounced RE(*f*)–O(*p*)–TM(*d*) orbital coupling engendered by Eu doping.

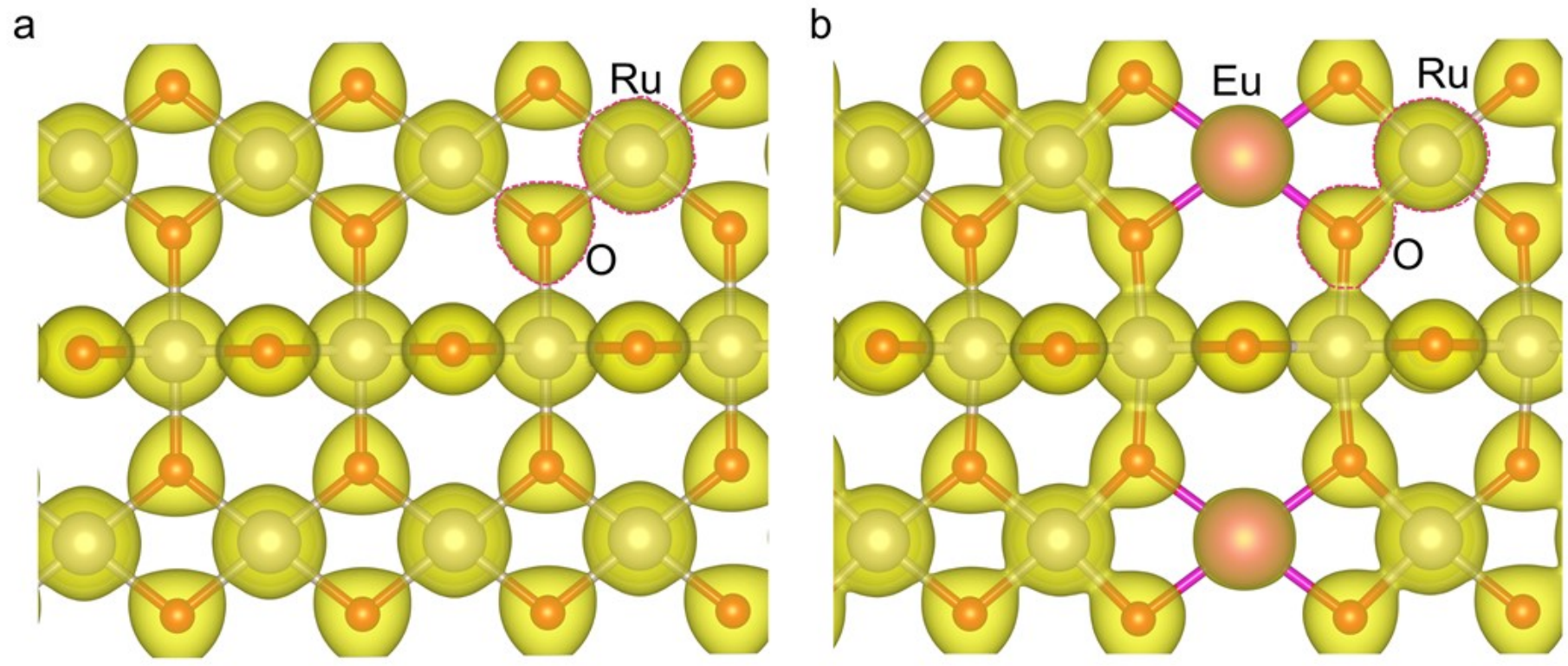

Fig. S15. Charge density images of RuO_2 (left) and Eu-RuO_2 (right).

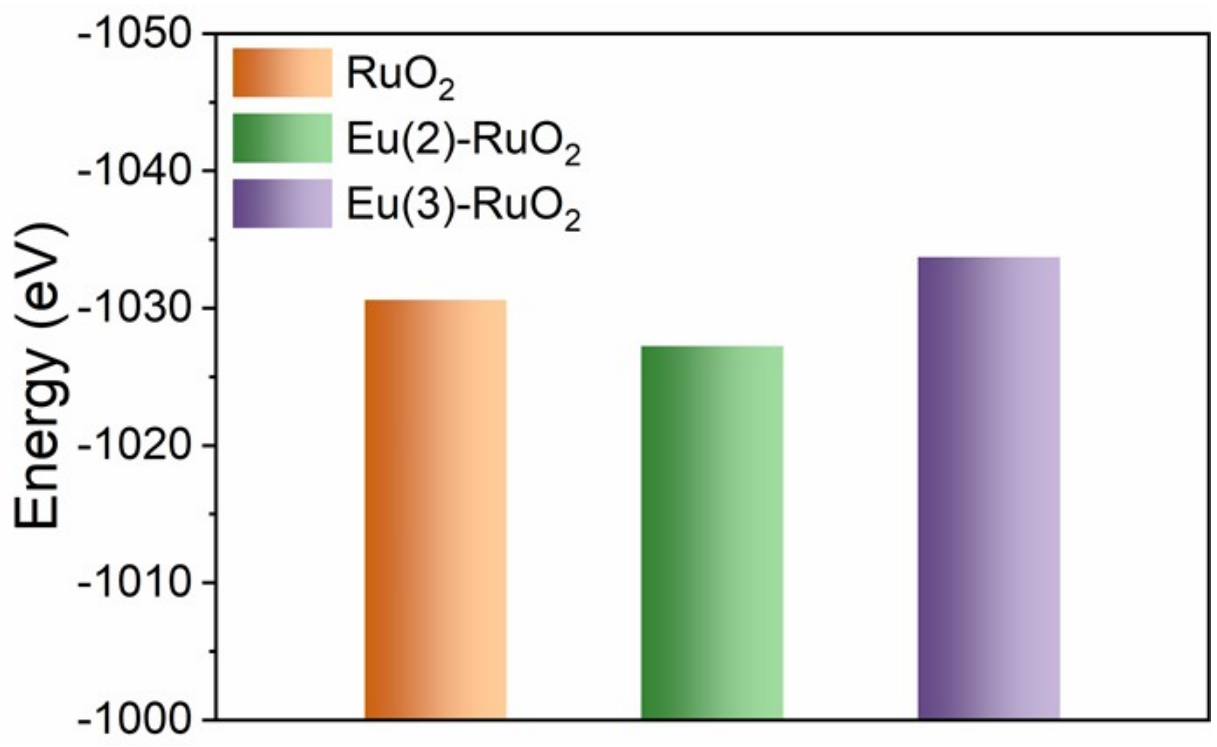

C

Fig. S16. Optimization energy levels of different valence states of Eu in Eu-RuO_2.

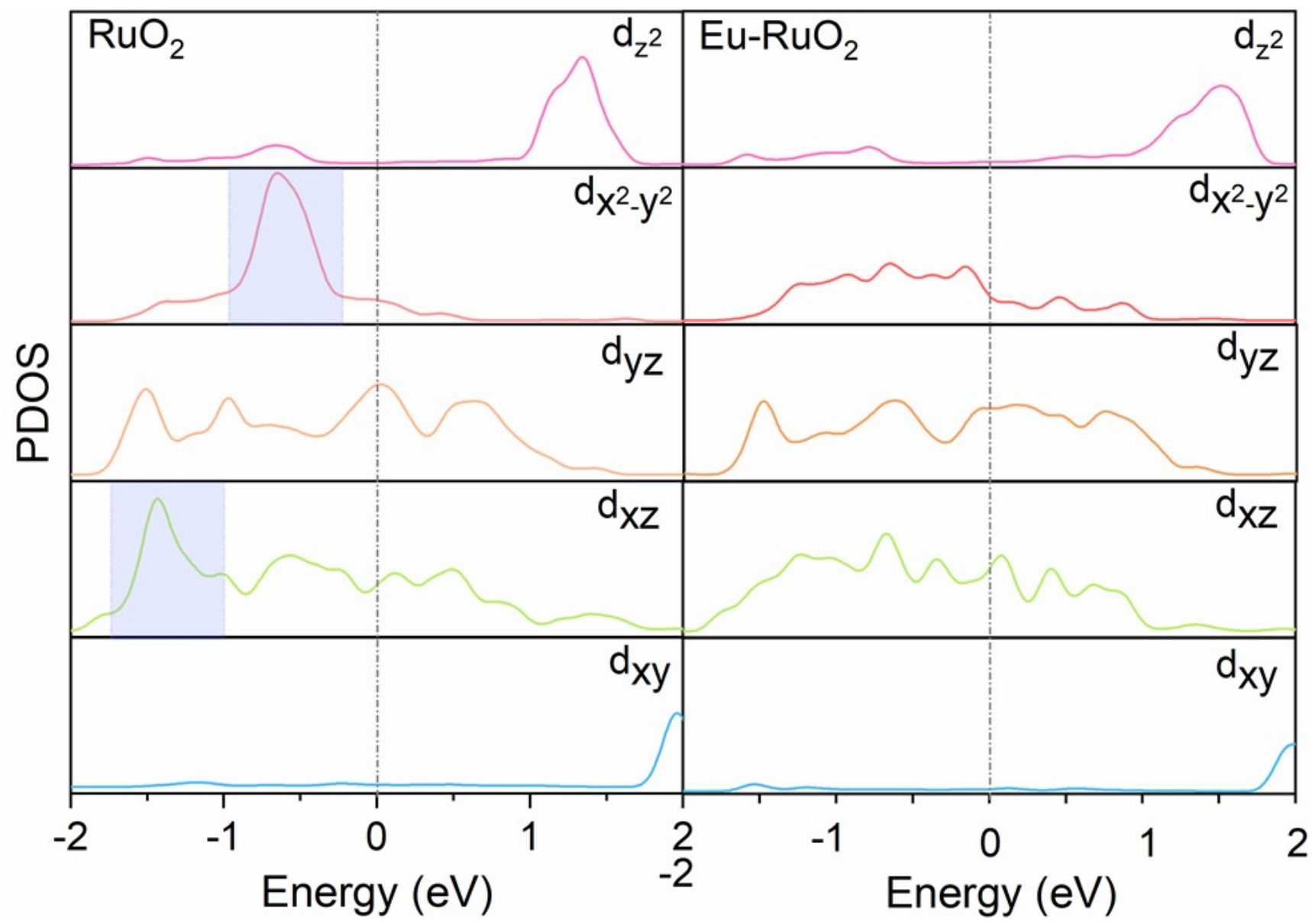

Fig. S17. The orbital-projected states of Ru *d* in RuO_2 and Eu-RuO_2.

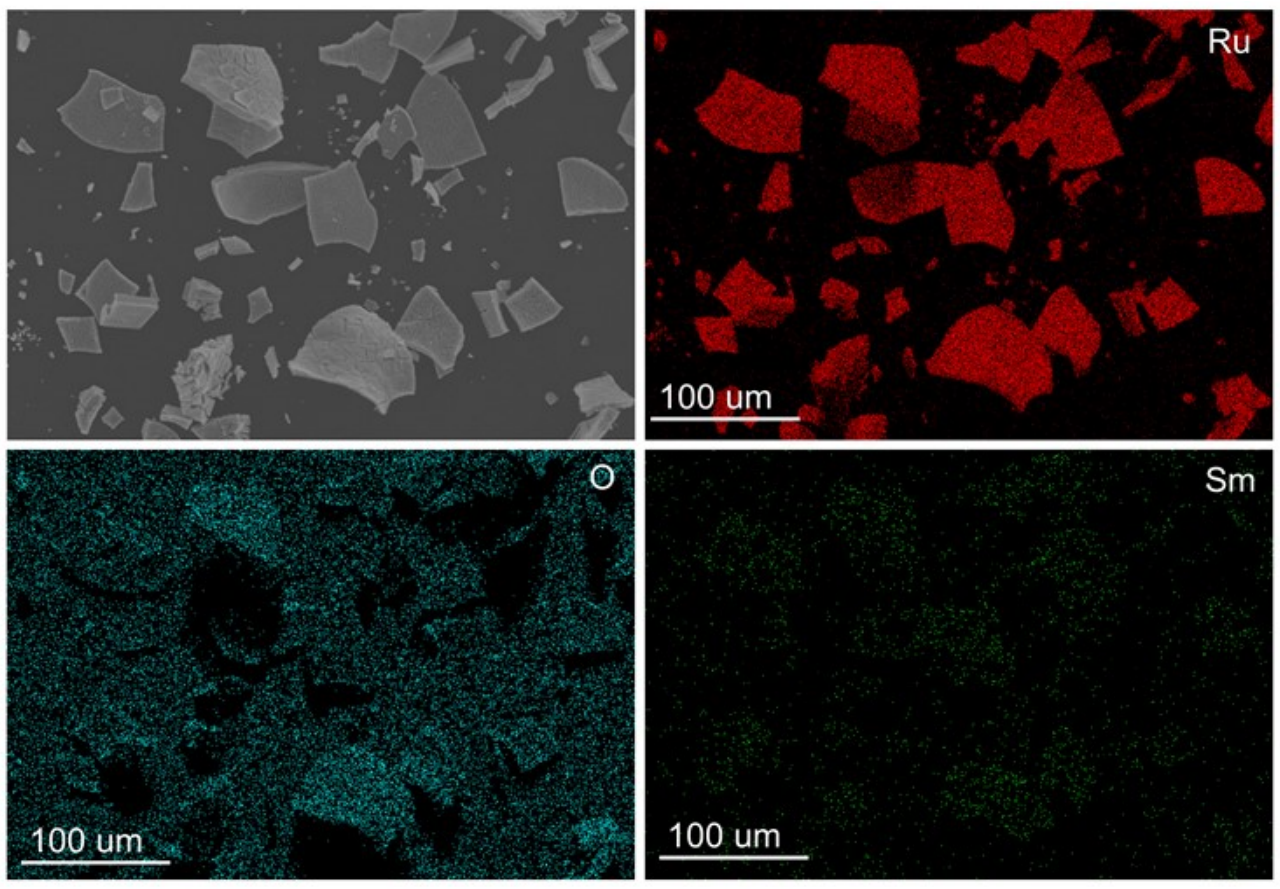

Fig. S18. SEM and corresponding elemental mapping images of Ru, Sm, and O for the Sm-RuO_2 sample.

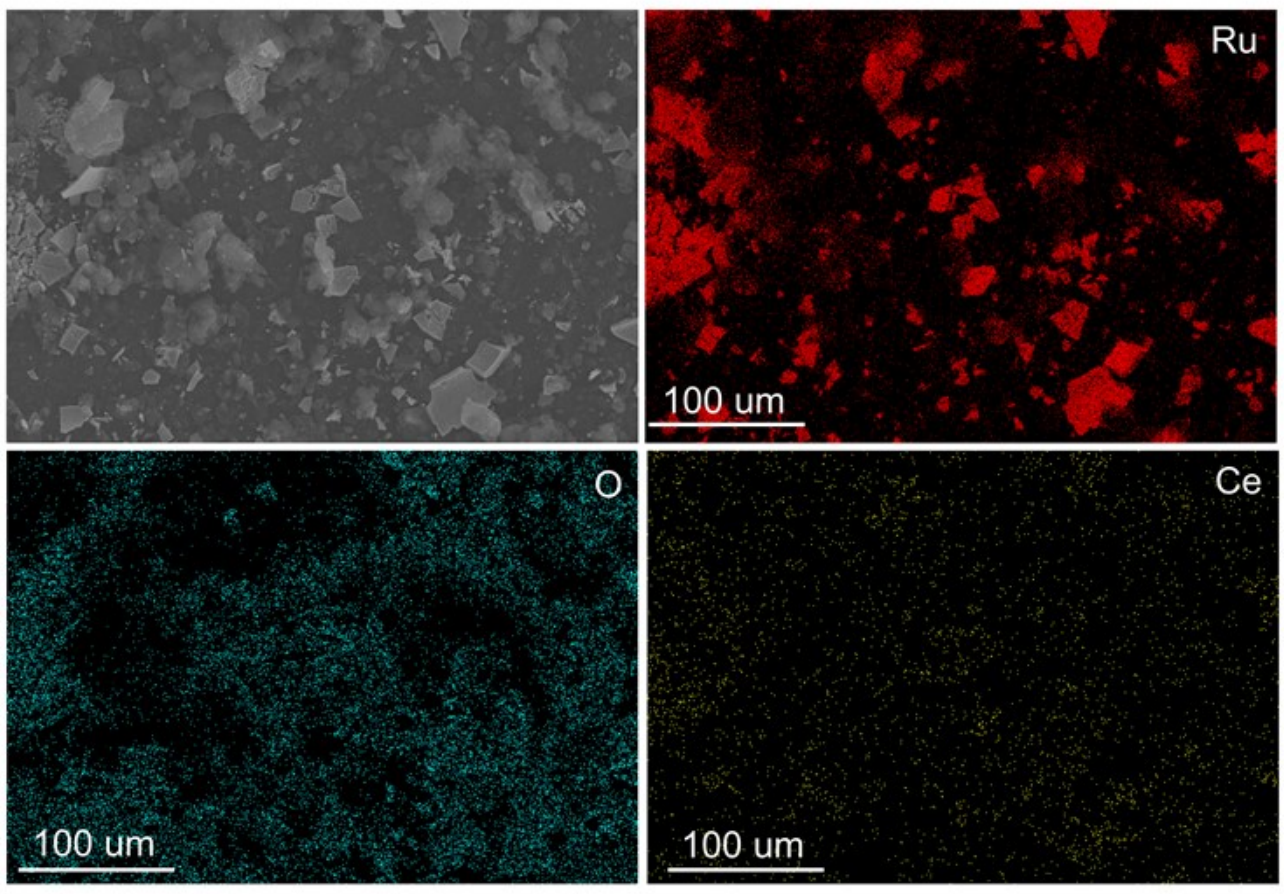

Fig. S19. SEM and corresponding elemental mapping images of Ru, Ce, and O for the $Ce\text{-}RuO_2$ sample.

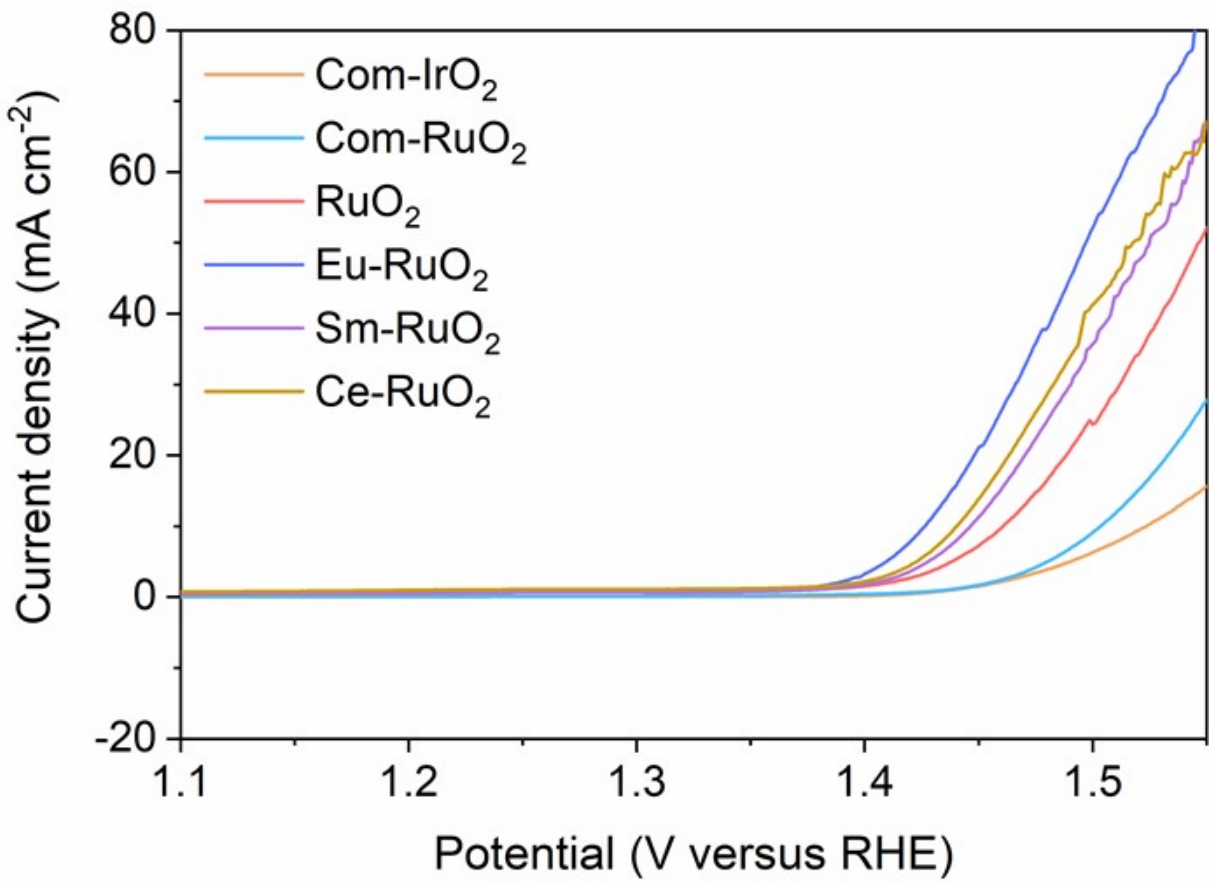

Fig. S20. The polarization curves tested at a scan rate of 5 mV s^{-1}.

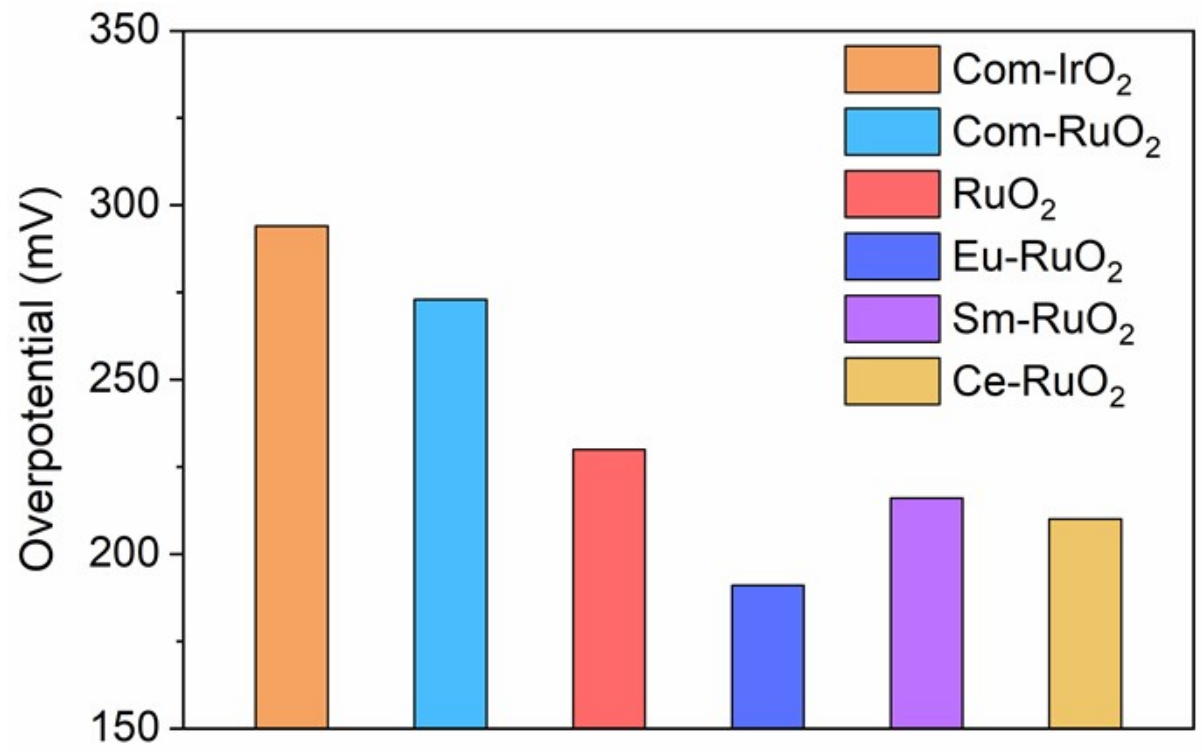

Fig. S21. The comparison of overpotentials at 10 mA cm^{-2}.

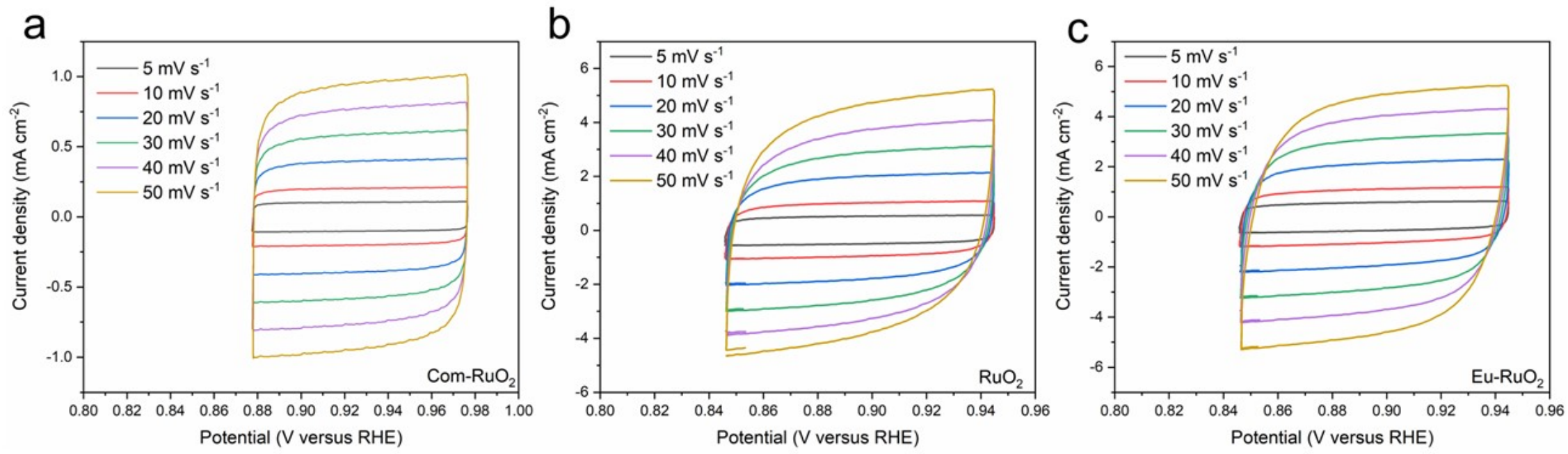

Fig. S22. The CV of commercial RuO_2, synthesized RuO_2 and Eu-RuO_2 at different scanning rate of 5, 10, 20, 30, 40 and 50 mV s^{-1}.

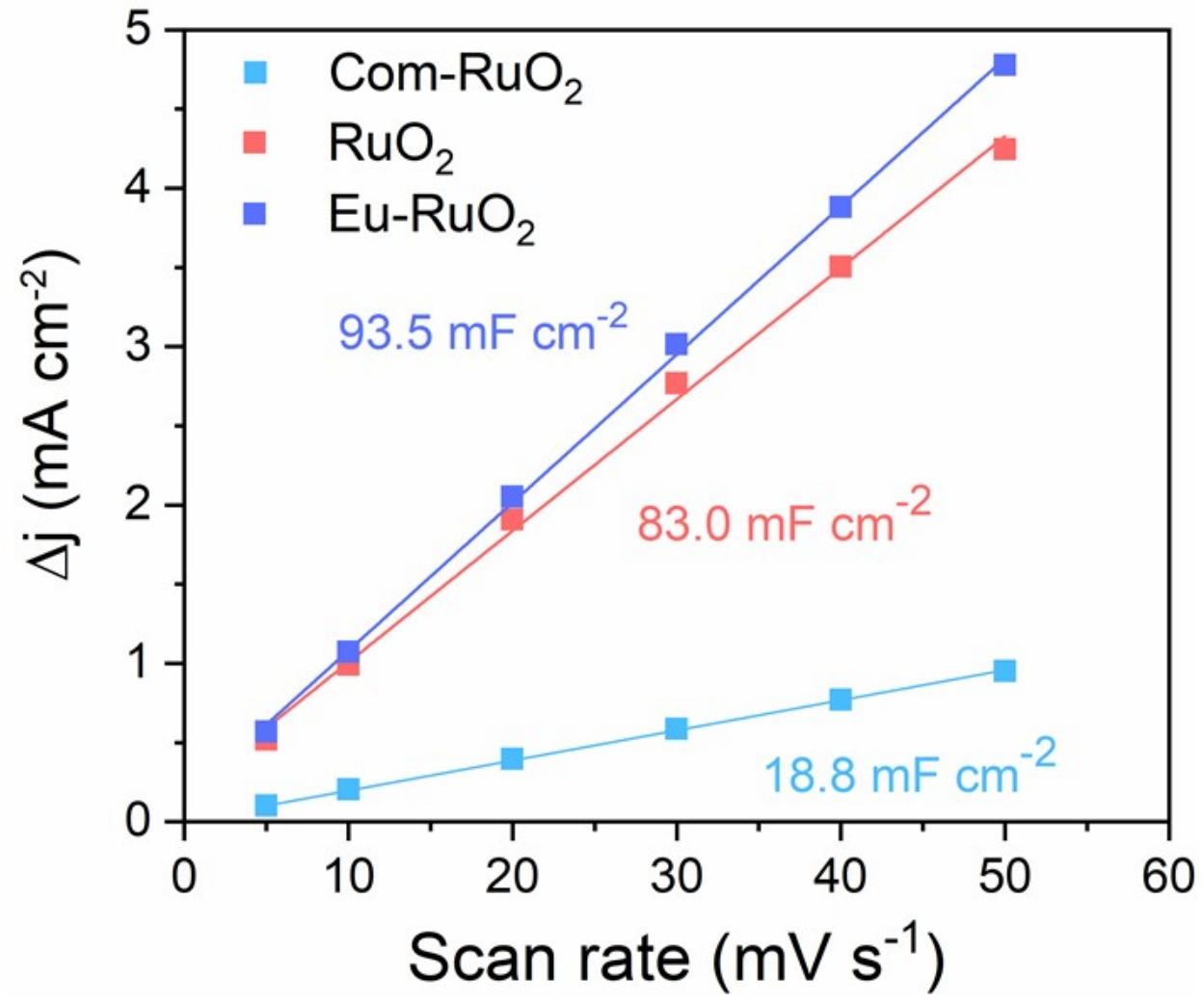

Fig. S23. The C_{dl} values of commercial RuO_2, synthesized RuO_2 and Eu-RuO_2.

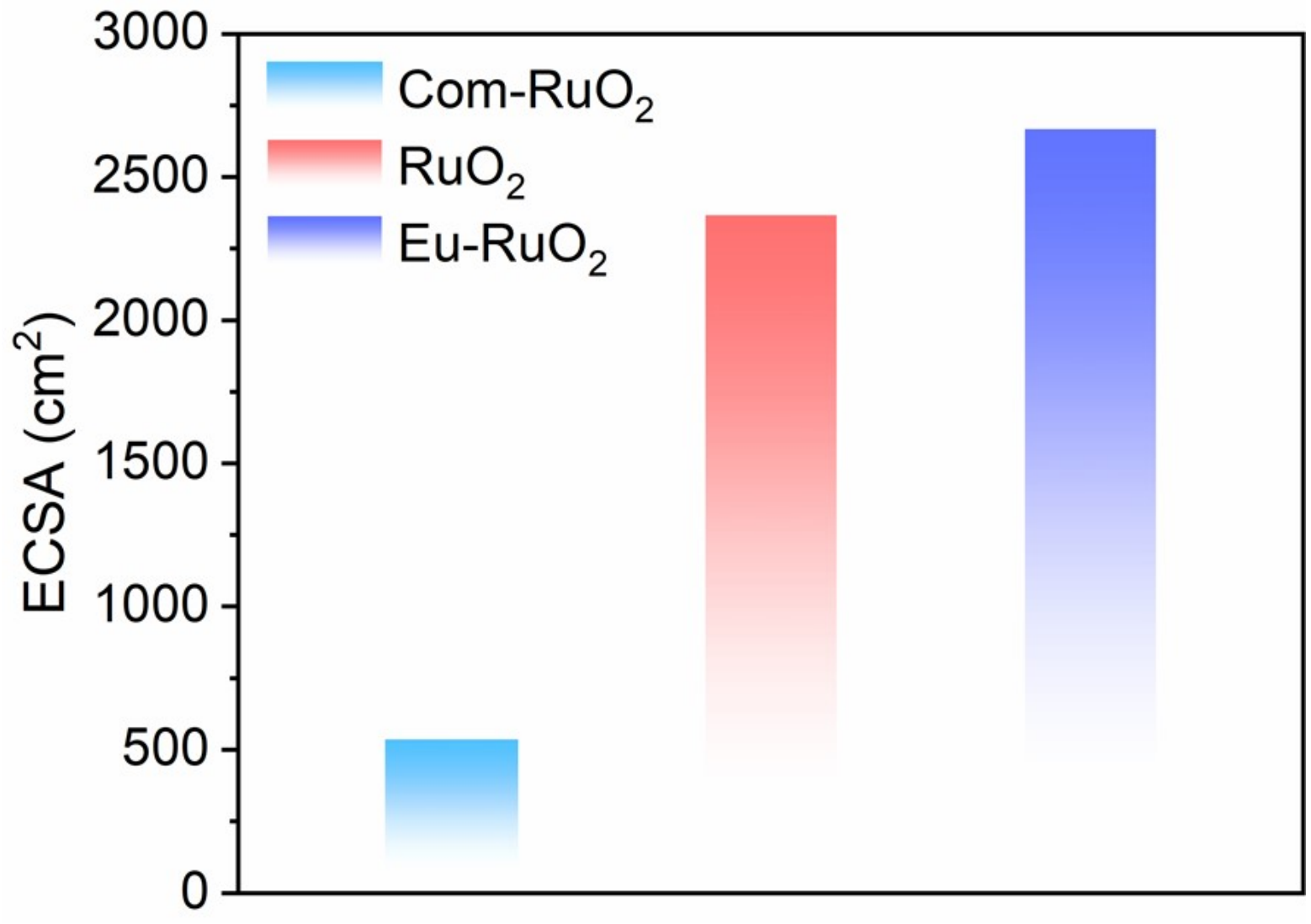

Fig. S24. The *ECSA* values of commercial RuO_2, synthesized RuO_2 and Eu-RuO_2.

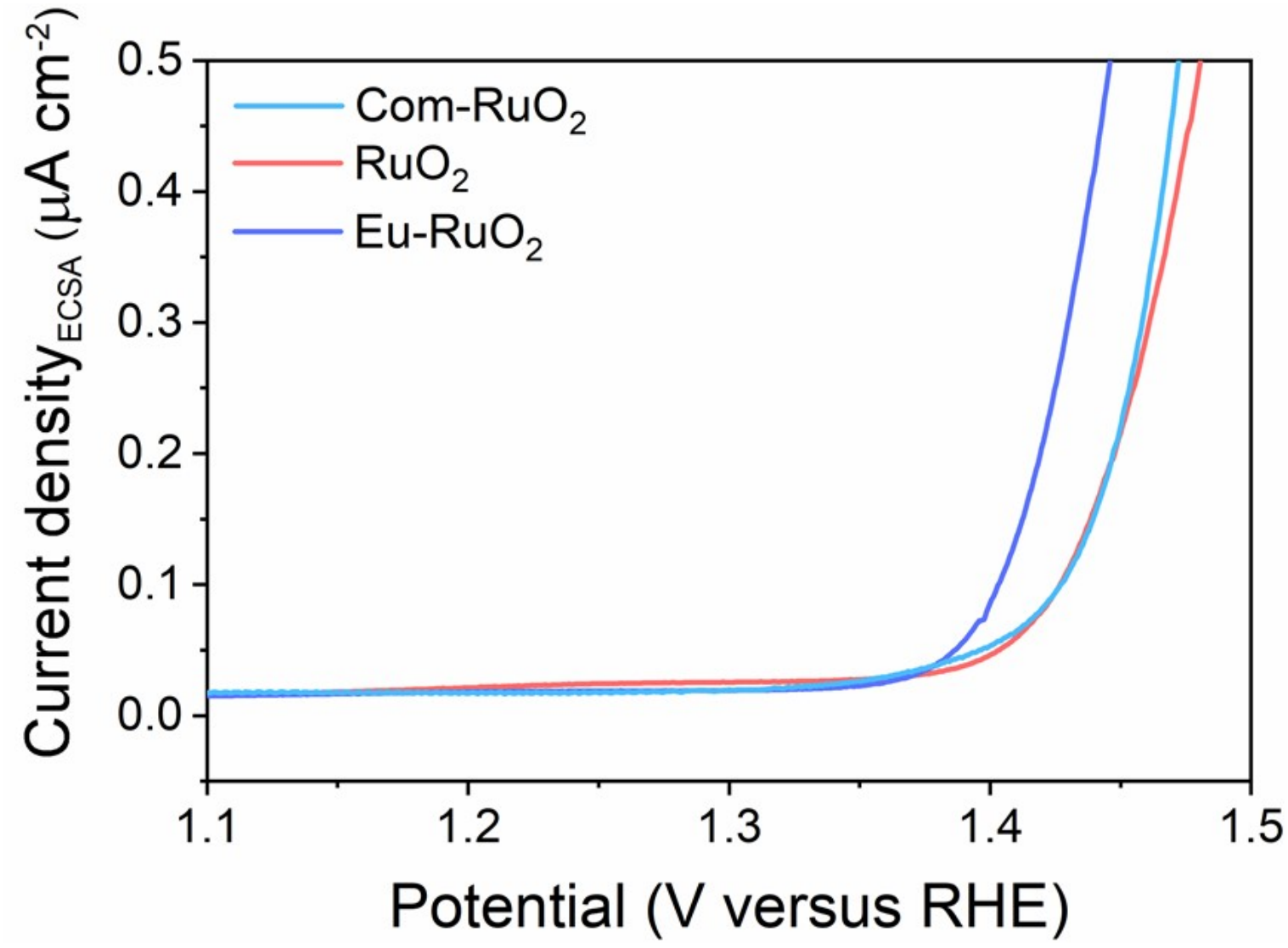

Fig. S25. The polarization curves based on *ECSA* values of commercial RuO_2, synthesized RuO_2 and Eu-RuO_2.

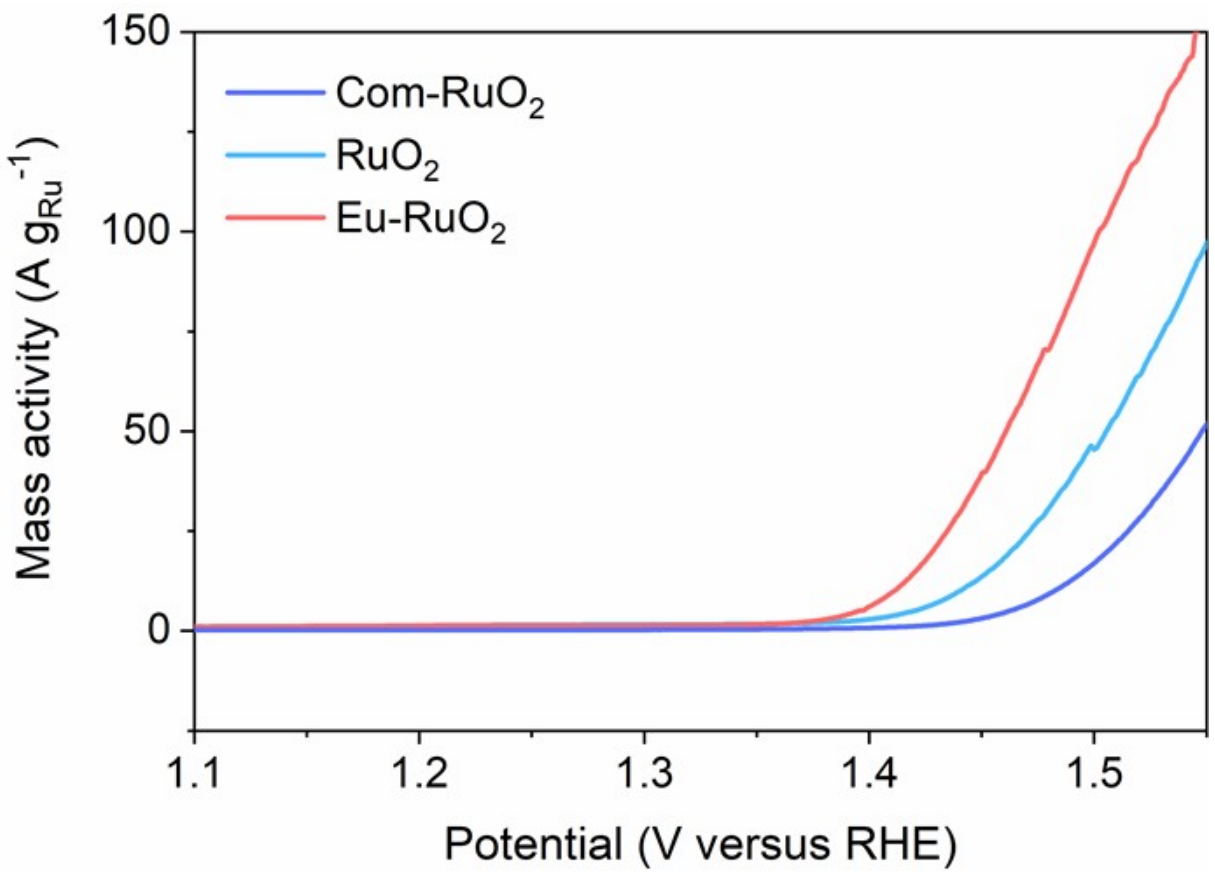

Fig. S26. The polarization curve from LSV test based on mass activity.

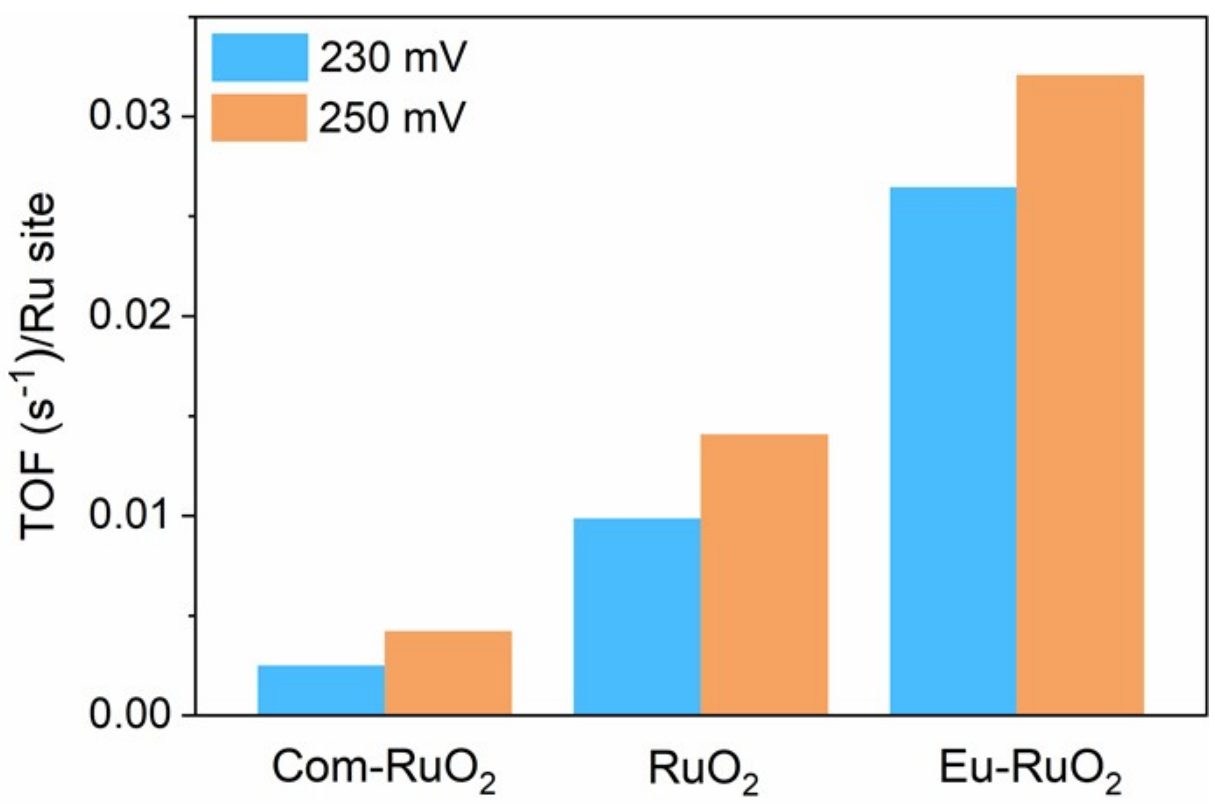

Fig. S27. Comparison of apparent TOF.

Table S2. Performance comparison of noble-metal based OER catalysts in acidic electrolyte.

Catalyst	substrate	Electrolyte	Overpotential at 10 mA cm^{-2} (mV)	Chronopotentiometry at specific current density	Ref.
Eu-RuO_2	GCE	0.5 M H_2SO_4	188	2800 h@10 mA cm^{-2}	This work
Co-Ru@RuO_2	GCE	0.5 M H_2SO_4	203	400 h@10 mA cm^{-2}	[12]
$W_{0.2}Er_{0.1}Ru_{0.7}O_{2-\delta}$	GCE	0.5 M H_2SO_4	168	500 h@10 mA cm^{-2}	[13]
$Li_{0.52}$RuO2	GCE	0.5 M H_2SO_4	156	70 h@10 mA cm^{-2}	[14]
Co-RuO_2	GCE	0.5 M H_2SO_4	169	50 h@10 mA cm^{-2}	[15]
$Cr_{0.6}Ru_{0.4}O_2$(550)	GCE	0.5 M H_2SO_4	178	10 h @10 mA cm^{-2}	[5]
$Ba_{0.3}(SO_4)_\delta W_{0.2}Ru_{0.5}O_{2-\delta}$	GCE	0.5 M H_2SO_4	188	1000 h @10 mA cm^{-2}	[16]
RuO_2 NSs	GCE	0.5 M H_2SO_4	199	5.56 h@10 mA cm^{-2}	[17]
Ru NPs	GCE	0.5 M H_2SO_4	202	10 h@10 mA cm^{-2}	[18]
Ru/MnO_2	GCE	0.5 M H_2SO_4	161	200 h@10 mA cm^{-2}	[19]
Ni-RuO_2	GCE	0.5 M H_2SO_4	214	200 h@10 mA cm^{-2}	[20]
Ru/RuO_2	GCE	0.5 M H_2SO_4	219	7 h @10 mA cm^{-2}	[21]
RuIrCo	Au	0.1 M $HClO_4$	235	25 h @10 mA cm^{-2}	[22]
E-Ru/Fe ONAs	GCE	0.5 M H2SO4	238	9 h @10 mA cm^{-2}	[23]
py-RuO_2: Zn	GCE	0.5 M H_2SO_4	173	1000 h@10 mA cm^{-2}	[24]
Pb-RuO_2	GCE	0.5 M H_2SO_4	188	1100 h @10 mA cm^{-2}	[4]
Na-RuO_2	GCE	0.5 M H_2SO_4	200	1800 h@10 mA cm^{-2}	[25]

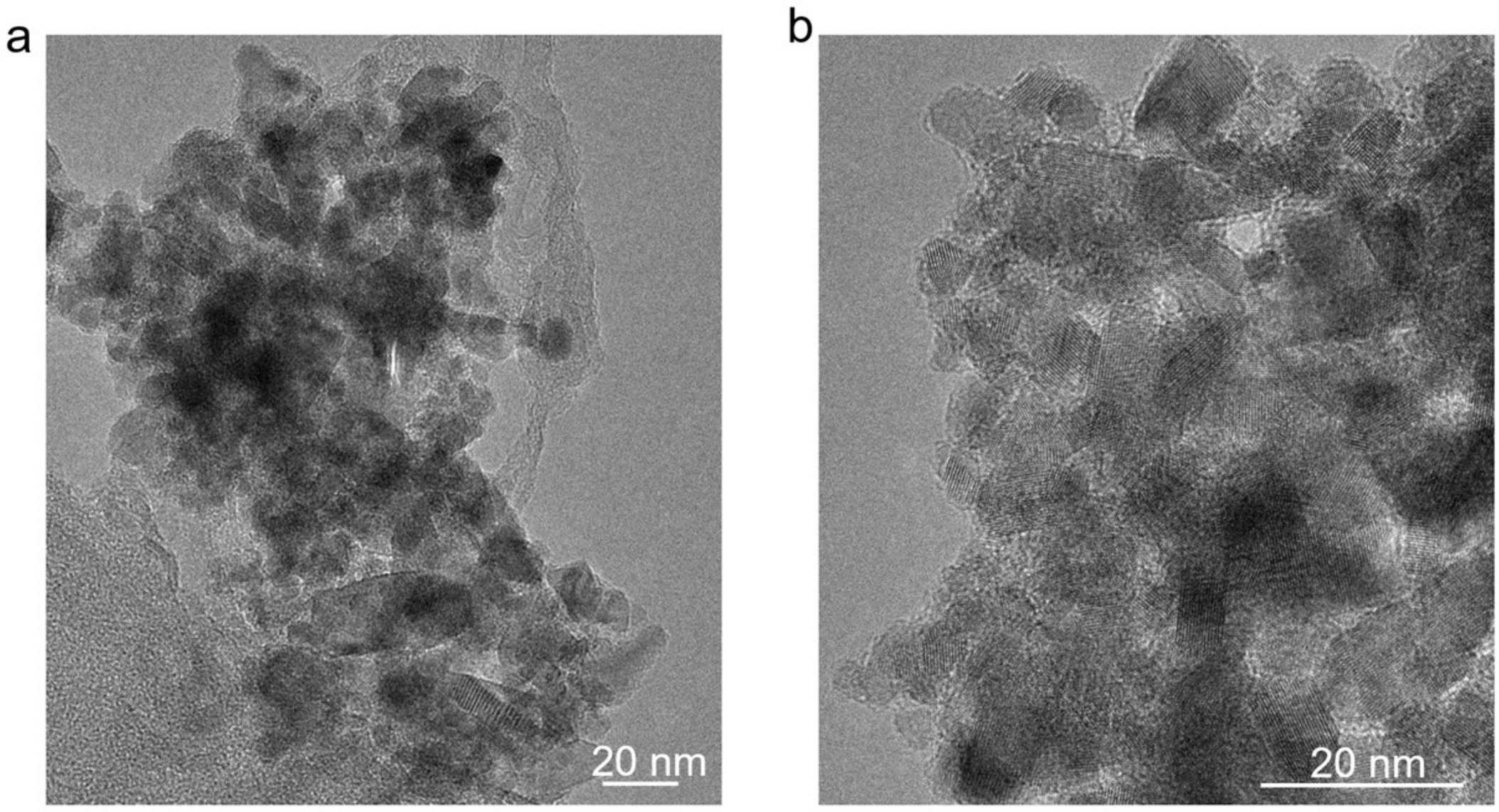

Fig. S28. HR-TEM image of the Eu-RuO_2 catalyst after OER.

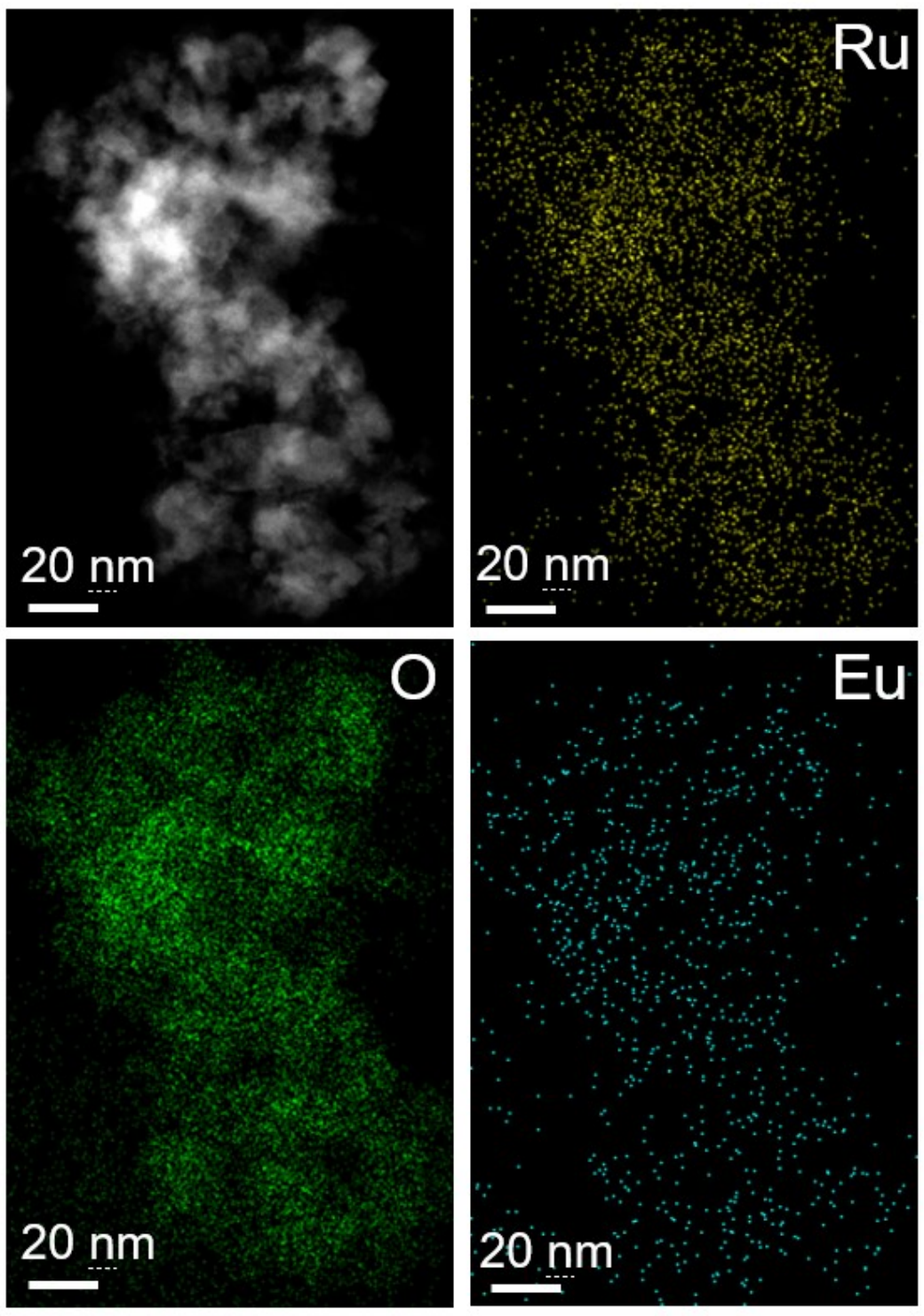

Fig. S29. Elemental mappings of Eu, Ru, and O in the Eu-RuO_2 catalyst after OER.

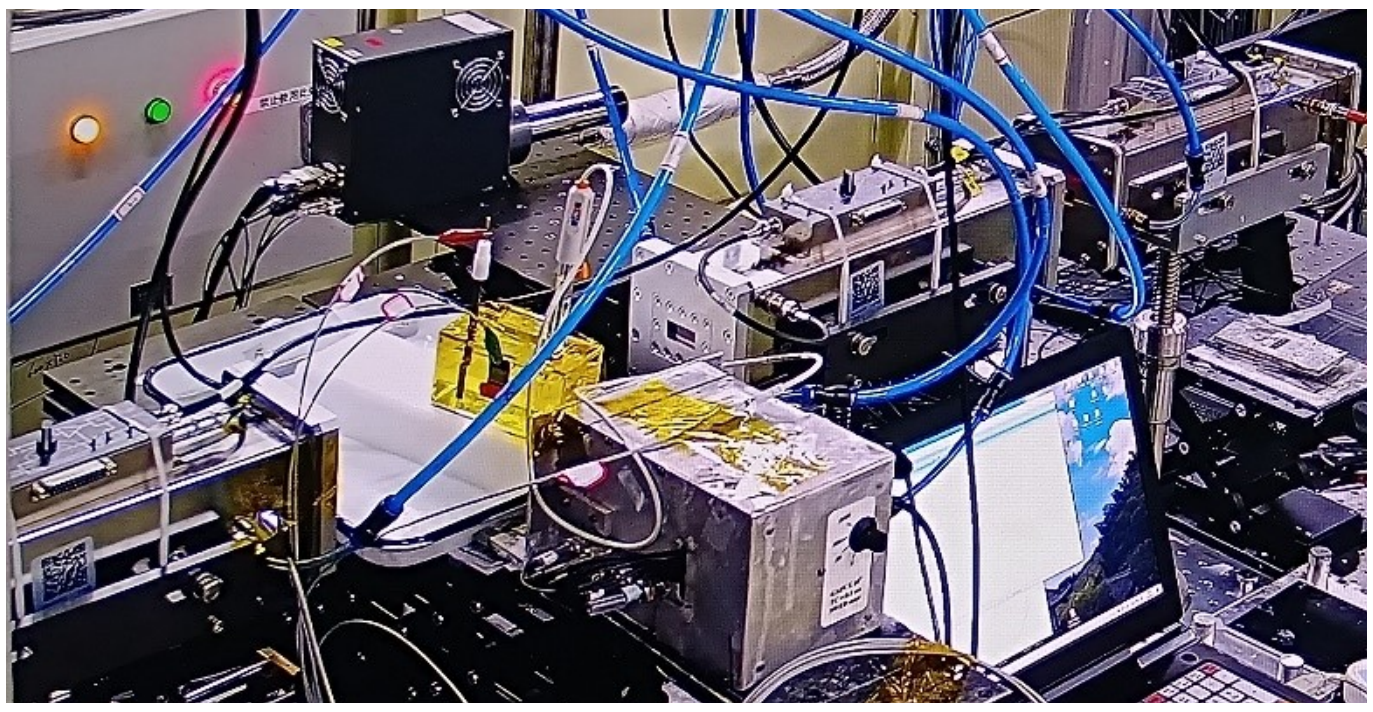

Fig. S30. Operando XAS measurements setup.

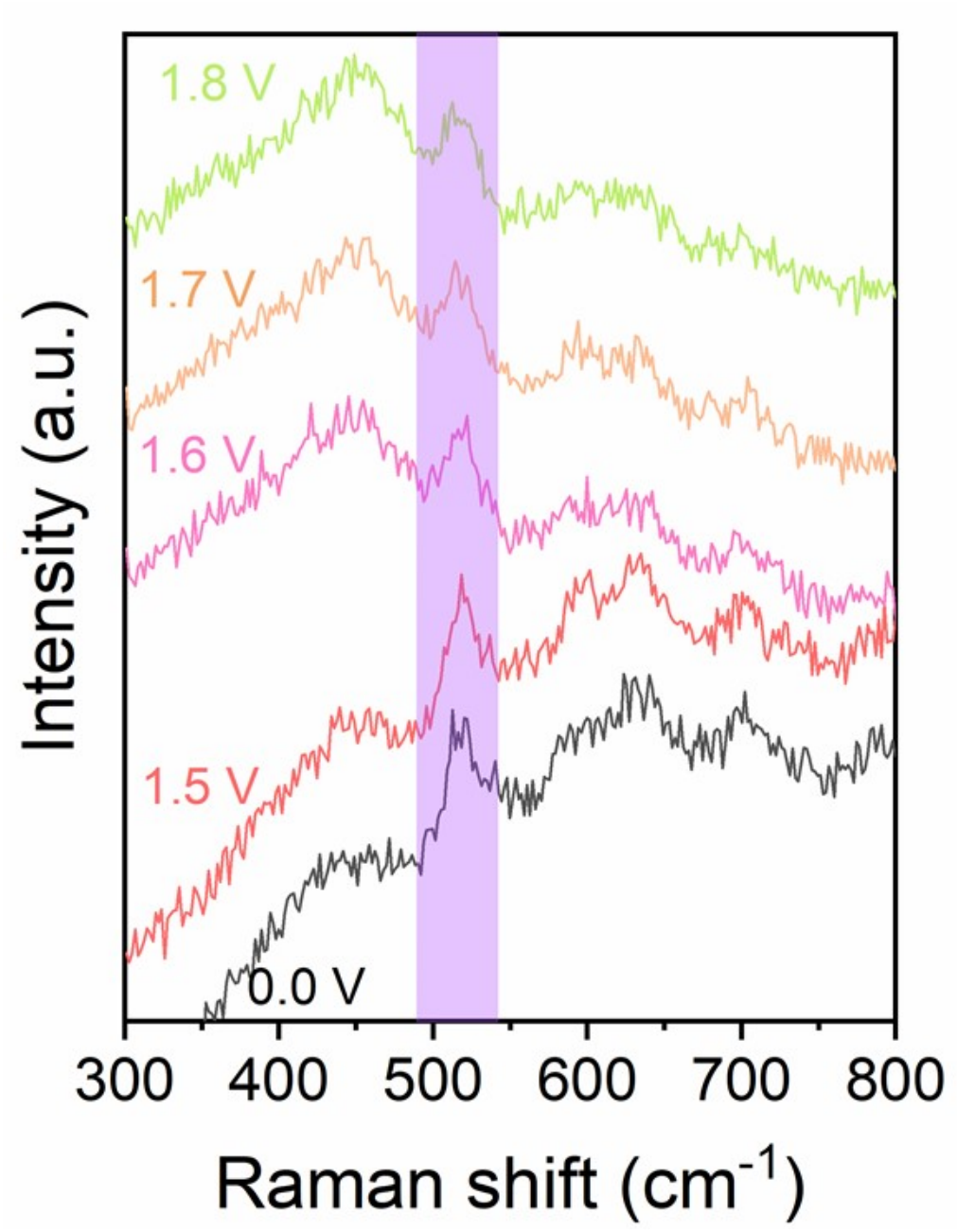

Fig. S31. Raman spectra of Eu-RuO_2 with different applied bias.

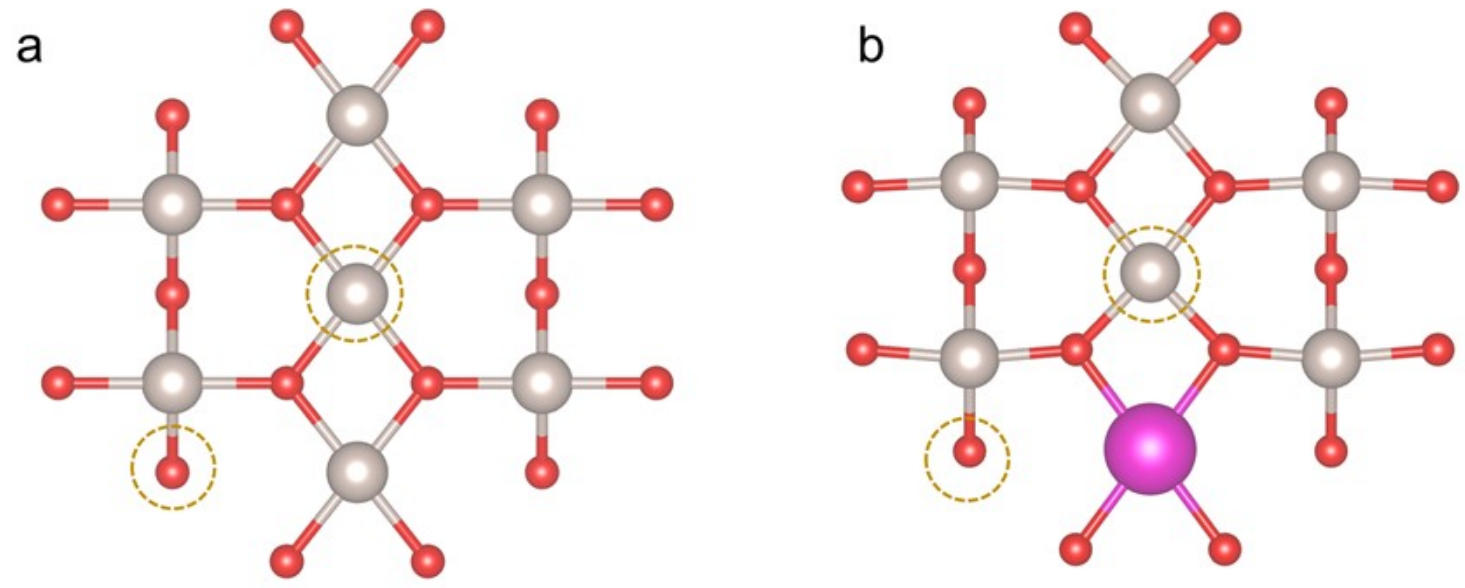

Fig. S32. DFT calculated sites for Ru and O vacancies.

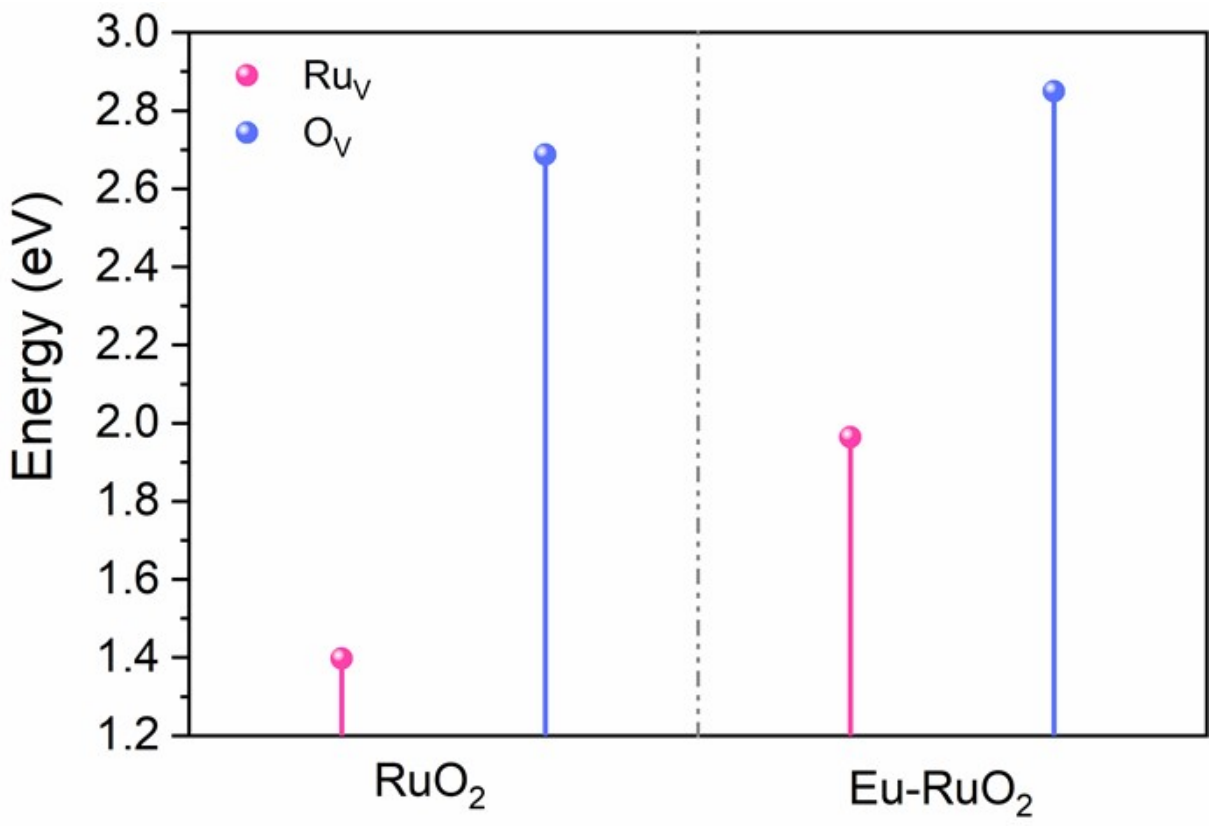

Fig. S33. Formation energies of Ru and O vacancies in RuO_2 and Eu-RuO_2.

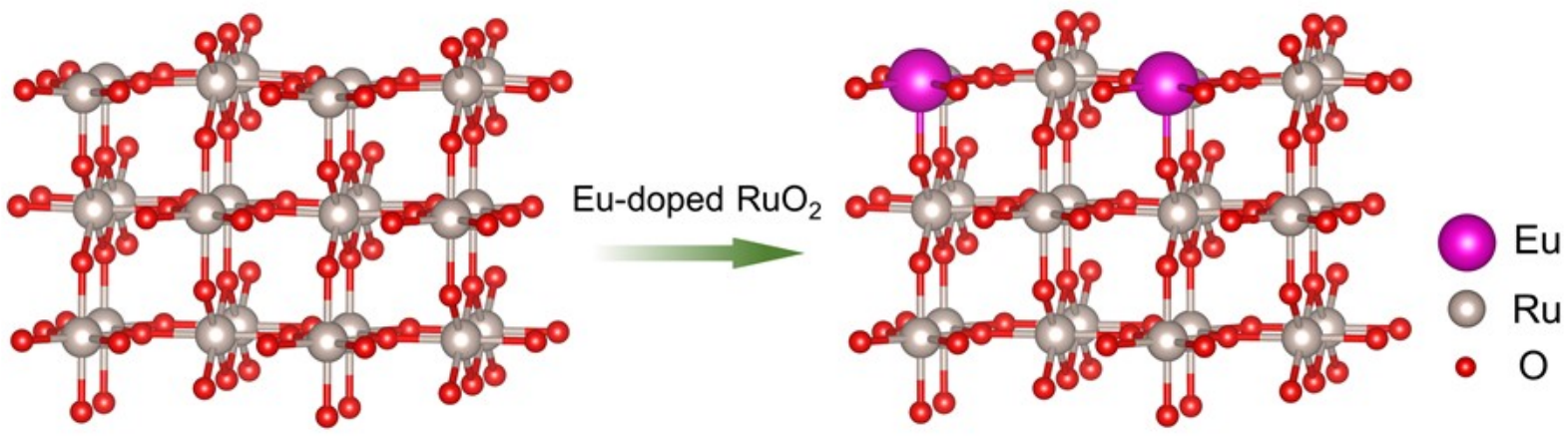

Fig. S34. The theoretical configurations of RuO_2 and Eu-doped RuO_2.

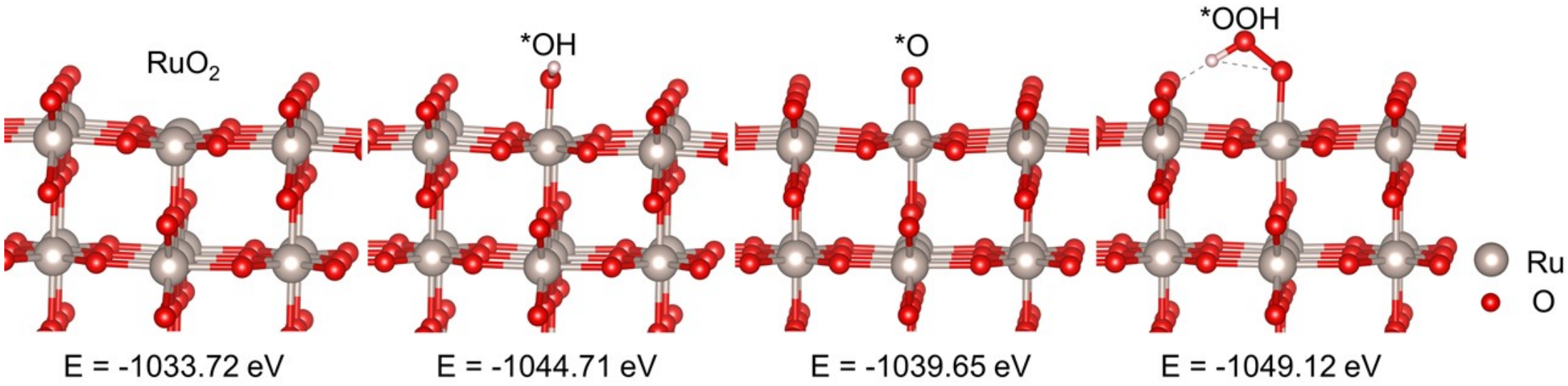

Fig. S35. The reaction paths of RuO_2 based on AEM.

Fig. S36. The reaction paths of Eu-RuO_2 based on AEM.

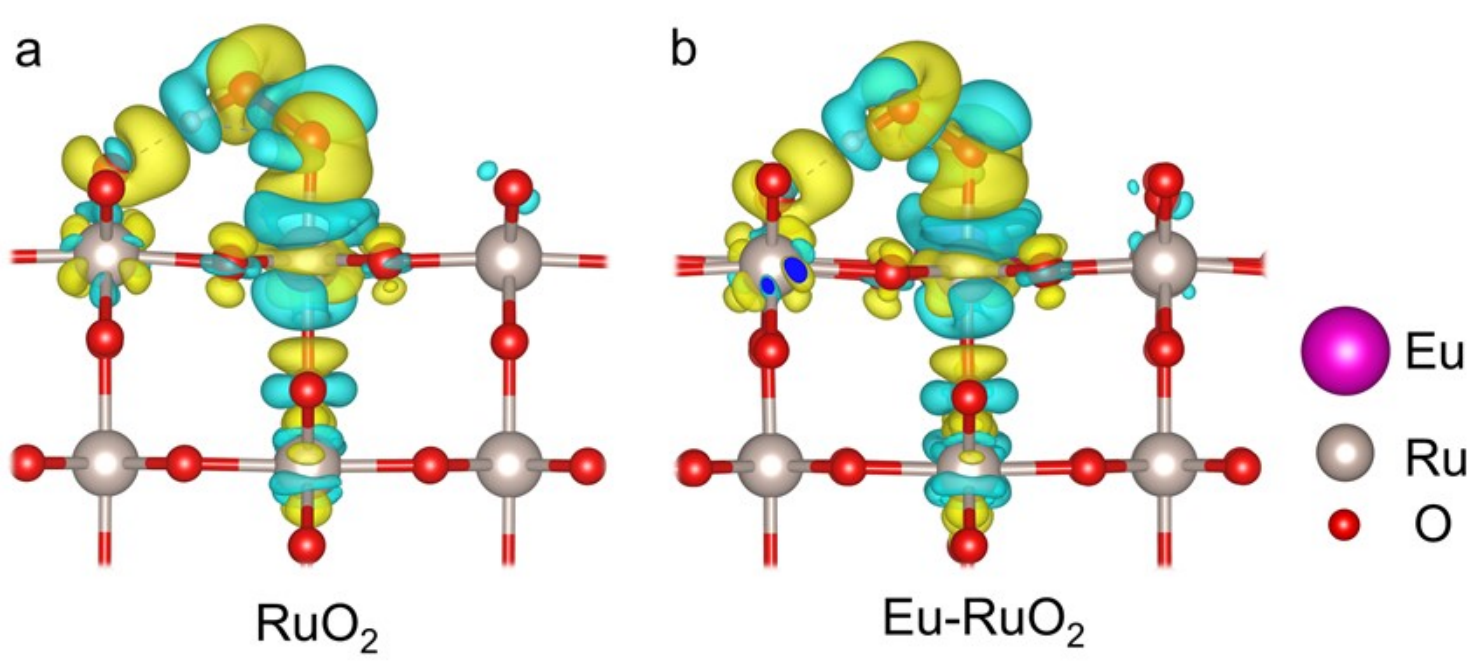

Fig. S37. Charge density difference of *OOH-adsorbed RuO_2 (a) and Eu-RuO_2 (b).

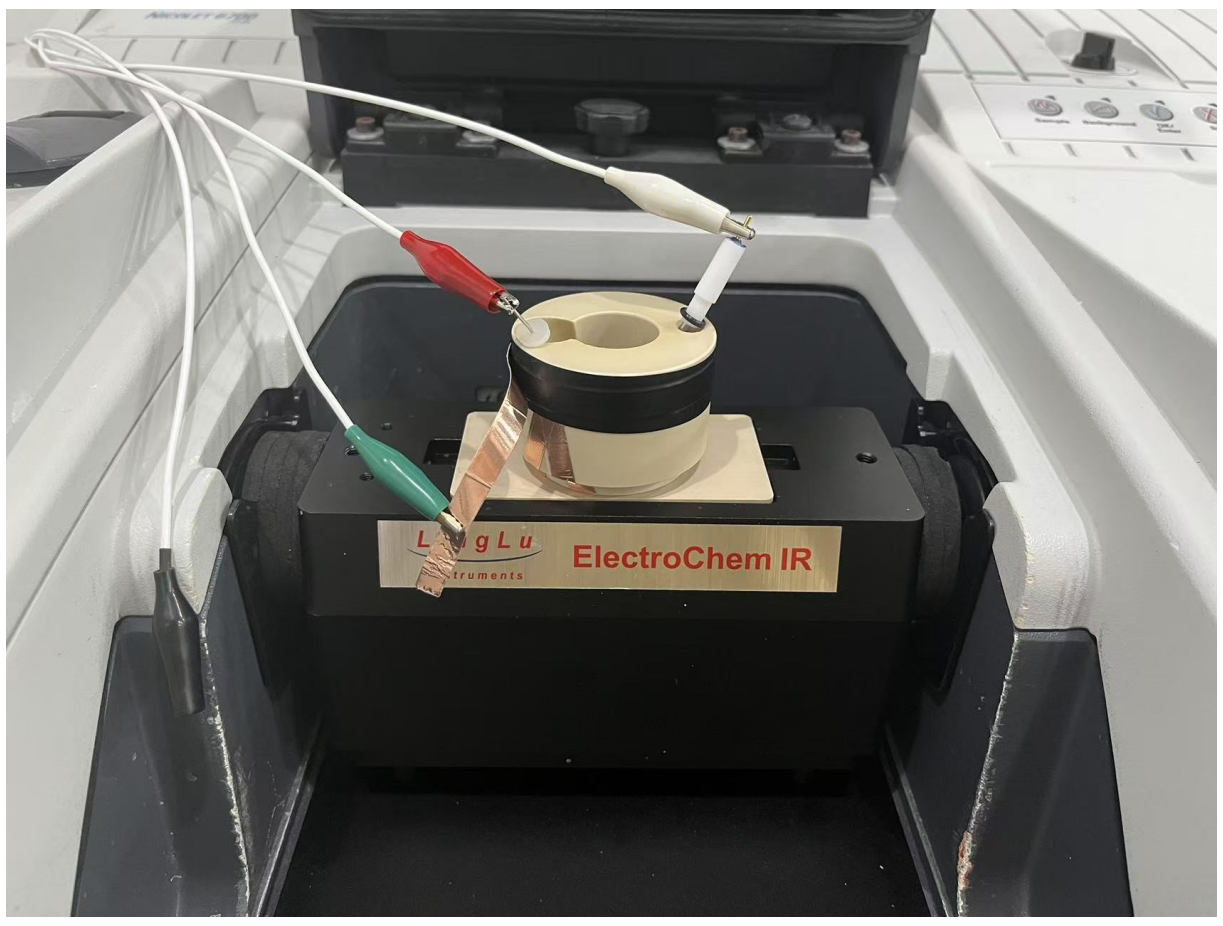

Fig. S38. Operando ATR-SEIRAS measurements setup.

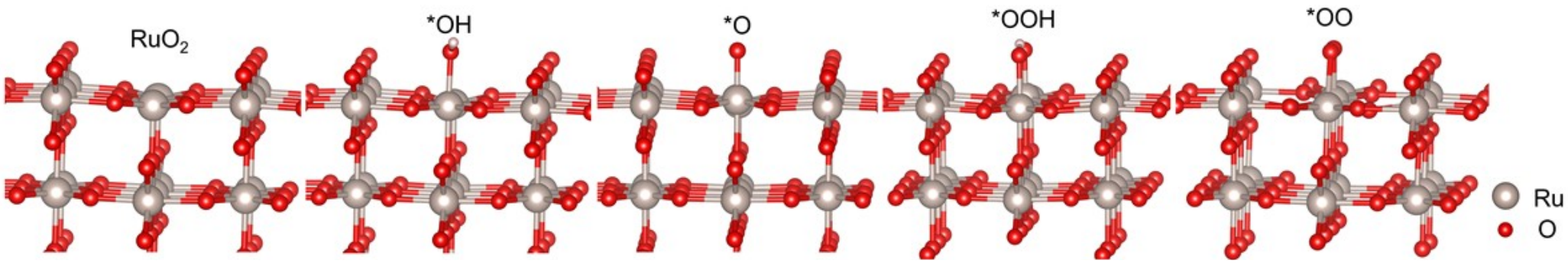

Fig. S39. The reaction paths of RuO_2 based on OPM.

Fig. S40. The reaction paths of Eu-RuO_2 based on OPM.

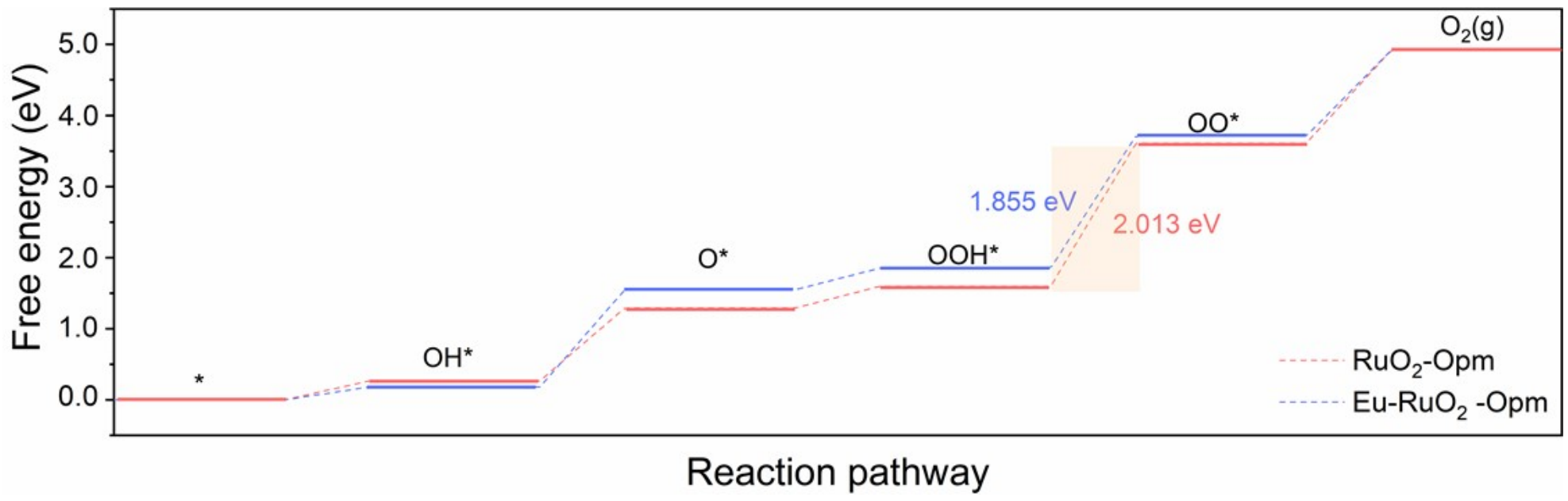

Fig. S41. Gibbs free energy diagrams of RuO_2 and Eu-RuO_2 based on OPM.

References

1. X. Wang, J. W. Wang, P. Wang, L. C. Li, X. Y. Zhang, D. M. Sun, Y. F. Li, Y. W. Tang, Y. Wang and G. T. Fu, *Adv Mater*, 2022, **34**.
2. M. Li, X. Wang, K. Liu, H. Sun, D. Sun, K. Huang, Y. Tang, W. Xing, H. Li and G. Fu, *Adv Mater*, 2023, **35**, e2302462.
3. J. van Elp, J. L. Wieland, H. Eskes, P. Kuiper, G. A. Sawatzky, F. M. de Groot and T. S. Turner, *Phys Rev B Condens Matter*, 1991, **44**, 6090-6103.
4. C. Zhou, L. Li, Z. Dong, F. Lv, H. Guo, K. Wang, M. Li, Z. Qian, N. Ye, Z. Lin, M. Luo and S. Guo, *Nat Commun*, 2024, **15**.
5. Y. Lin, Z. Tian, L. Zhang, J. Ma, Z. Jiang, B. J. Deibert, R. Ge and L. Chen, *Nat Commun*, 2019, **10**, 162.
6. S. Kozuch and J. M. L. Martin, *ACS Catalysis*, 2012, **2**, 2787-2794.
7. L. Li, G. W. Zhang, C. H. Zhou, F. Lv, Y. J. Tan, Y. Han, H. Luo, D. W. Wang, Y. X. Liu, C. S. Shang, L. Y. Zeng, Q. Z. Huang, R. J. Zeng, N. Ye, M. C. Luo and S. J. Guo, *Nat Commun*, 2024, **15**.
8. G. Kresse and J. Furthmuller, *Comp Mater Sci*, 1996, **6**, 15-50.
9. G. Kresse and J. Furthmuller, *Phys Rev B*, 1996, **54**, 11169-11186.
10. J. P. Perdew, K. Burke and M. Ernzerhof, *Phys Rev Lett*, 1996, **77**, 3865-3868.
11. G. Kresse and D. Joubert, *Phys Rev B*, 1999, **59**, 1758-1775.

12. J. Chen, Y. Ma, C. Cheng, T. Huang, R. Luo, J. Xu, X. Wang, T. Jiang, H. Liu, S. Liu, T. Huang, L. Zhang and W. Chen, *J Am Chem Soc*, 2025, **147**, 8720-8731.
13. S. Hao, M. Liu, J. Pan, X. Liu, X. Tan, N. Xu, Y. He, L. Lei and X. Zhang, *Nat Commun*, 2020, **11**, 118605.
14. Y. Qin, T. Yu, S. Deng, X. Y. Zhou, D. Lin, Q. Zhang, Z. Jin, D. Zhang, Y. B. He, H. J. Qiu, L. He, F. Kang, K. Li and T. Y. Zhang, *Nat Commun*, 2022, **13**, 3784.
15. Y. Tian, S. Wang, E. Velasco, Y. Yang, L. Cao, L. Zhang, X. Li, Y. Lin, Q. Zhang and L. Chen, *Iscience*, 2020, **23**, 100756.
16. Y. R. Xue, J. W. Zhao, L. Huang, Y. R. Lu, A. Malek, G. Gao, Z. B. Zhuang, D. S. Wang, C. T. Yavuz and X. Lu, *Nat Commun*, 2023, **14**.
17. Z. L. Zhao, Q. Wang, X. Huang, Q. Feng, S. Gu, Z. Zhang, H. Xu, L. Zeng, M. Gu and H. Li, *Energy Environ Sci*, 2020, **13**, 5143-5151.
18. J.-Q. Wang, C. Xi, M. Wang, L. Shang, J. Mao, C.-K. Dong, H. Liu, S. A. Kulinich and X.-W. Du, *ACS Catal*, 2020, **10**, 12575-12581.
19. C. Lin, J.-L. Li, X. Li, S. Yang, W. Luo, Y. Zhang, S.-H. Kim, D.-H. Kim, S. S. Shinde, Y.-F. Li, Z.-P. Liu, Z. Jiang and J.-H. Lee, *Nat Catal*, 2021, **4**, 1012-1023.
20. Z. Y. Wu, F. Y. Chen, B. Li, S. W. Yu, Y. Z. Finfrock, D. M. Meira, Q. Q. Yan, P. Zhu, M. X. Chen, T. W. Song, Z. Yin, H. W. Liang, S. Zhang, G. Wang and H. Wang, *Nat Mater*, 2022, **22**, 100-108.
21. Z. Wang, B. Xiao, Z. Lin, S. Shen, A. Xu, Z. Du, Y. Chen and W. Zhong, *J Energy Chem*, 2021, **54**, 510-518.
22. J. Shan, T. Ling, K. Davey, Y. Zheng and S.-Z. Qiao, *Adv Mater*, 2019, **31**.
23. Q. Yao, B. Huang, Y. Xu, L. Li, Q. Shao and X. Huang, *Nano Energy*, 2021, **84**, 105909.
24. D. Zhang, M. Li, X. Yong, H. Song, G. I. N. Waterhouse, Y. Yi, B. Xue, D. Zhang, B. Liu and S. Lu, *Nat Commun*, 2023, **14**, 2517.
25. W. Li, D. Chen, Z. Lou, H. Yuan, X. Fu, H. Y. Lin, M. Lin, Y. Hou, H. Qi, P. F. Liu, H. G. Yang and H. Wang, *J Am Chem Soc*, 2025, DOI: 10.1021/jacs.4c18300.
